# Causal relationship between gut microbiota and myasthenia gravis: a two-sample Mendelian randomization study

**DOI:** 10.3389/fneur.2024.1309530

**Published:** 2024-01-25

**Authors:** Chuanhao Mi, Ajiao Hou, Ziyue Wang, Xianghua Qi, Jing Teng

**Affiliations:** ^1^First Clinical Medical College, Shandong University of Traditional Chinese Medicine, Jinan, Shandong, China; ^2^Key Laboratory of Basic and Application Research of Beiyao, Heilongjiang University of Chinese Medicine, Harbin, Heilongjiang, China; ^3^Department of Neurology, Affiliated Hospital of Shandong University of Traditional Chinese Medicine, Jinan, Shandong, China

**Keywords:** genome-wide association study, myasthenia gravis, causal relationship, gut microbiota, Mendelian randomization

## Abstract

**Background:**

Previous observational studies have provided cumulative data linking gut microbiota to myasthenia gravis (MG). However, the causal link between the two remains unexplored. Hence, the current study was performed to explore the causal link between them.

**Methods:**

Mendelian randomization (MR) analysis was conducted using the summary statistics of 211 gut microbiota taxa and the largest genome-wide association studies (GWAS) for MG currently available. The inverse variance-weighted (IVW), MR-Egger, weighted median, and weighted mode methods were employed to ascertain the causal influence. Sensitivity studies utilizing several methodologies were then used to assess the robustness of the findings. Lastly, to evaluate reverse causality, a reverse MR analysis was performed.

**Results:**

Seven suggestive causal associations between the gastrointestinal microbiota and MG were identified based on the outcomes of the MR analysis. Specifically, phylum Actinobacteria (OR: 0.602, 95% CI: 0.405–0.896, *p* = 0.012), class Gammaproteobacteria (OR: 0.587, 95% CI: 0.357–0.968, *p* = 0.037), and families *Defluviitaleaceae* (OR: 0.695, 95% CI: 0.485–0.996, *p* = 0.047), *Family XIII* (OR: 0.614, 95% CI: 0.412–0.916, *p* = 0.017), and *Peptococcaceae* (OR: 0.698, 95% CI: 0.505–0.964, *p* = 0.029) had suggestive protective effects on MG, while order Mollicutes RF9 (OR: 1.424, 95% CI: 1.015–1.998, *p* = 0.041) and genus *Faecalibacterium* (OR: 1.763, 95% CI: 1.220–2.547, *p* = 0.003) were suggestive risk factors for MG. The outcomes indicate that neither heterogeneity nor horizontal pleiotropy had any discernible impact. Nevertheless, this reverse analysis did not reveal any apparent effect of MG on the gut microbiota composition.

**Conclusion:**

The MR investigation has substantiated the suggestive causal connection between gut microbiota and MG, which may provide helpful insights for innovative therapeutic and preventative approaches for MG. Further randomized controlled trials are needed to elucidate the gut microbiota’s precise role and therapeutic potential in the pathogenesis of MG.

## 1 Introduction

The autoimmune disease myasthenia gravis (MG), characterized by autoantibody-mediated impairment of neuronal transmission at the neuromuscular junction (NMJ), has an incidence and prevalence of minimal regional variation. Epidemiological investigations have indicated that the global prevalence of MG ranges from 40 to 180 cases per million individuals, while the yearly incidence falls within the range of 4–12 cases per million individuals (1). According to statistical data, it has been shown that around 80% of patients diagnosed with MG have acetylcholine receptor (AChR) antibody-positive. Conversely, a smaller proportion of individuals have muscle-specific kinase (MuSK) antibody-positive or lipoprotein receptor-related protein 4 (LRP4) antibody-positive as well (2). Fatigue and partial or systemic muscle weakness are caused by the NMJ being degraded by these antibodies. These symptoms serve as the primary clinical indicators of MG (3, 4). Patients diagnosed with MG are categorized into two main subtypes, ocular myasthenia gravis (OMG) and generalized myasthenia gravis (GMG), based on the specific muscles that are affected (5). Approximately 30–80% of individuals diagnosed with OMG are likely to progress to GMG over a span of 2 years (6). In addition to ptosis and diplopia, these people exhibit bulbar symptoms, limb weakness, and possibly respiratory failure (4). Hence, prompt detection and management through novel therapeutic interventions is critical for the treatment of such individuals.

The gut microbiota, a highly intricate and dynamic microbial population, resides within the gastrointestinal system of humans. Over the past decades, there has been a significant surge in scientific interest surrounding gut microbiota due to its strong association with immunology, inflammation, nutritional intake, and a wide range of disorders (7, 8). Emerging scientific evidence shows that the gastrointestinal microbiota greatly influences the development and progression of MG. Multiple case–control studies conducted on individuals with MG have shown alterations in the gut microbiota composition upon comparison to a healthy control group. These alterations primarily manifest as changes in the relative abundances of specific bacterial taxa (9, 10). An animal experiment using fecal microbiota transplantation (FMT) showed that, after immunized using the same classical MG modeling approach (experimental autoimmune MG) (11), MG microbiome mice (MMb mice, germ-free mice colonized with the fecal samples from MG patients) resulted in significant impaired motor ability, such as shorter total distance traveled in open field test (OFT), compared to healthy microbiota mice (HMb mice, germ-free mice colonized with the fecal samples from healthy controls). However, this effect could reversed by colonizing with the fecal samples from MG patients and healthy controls in germ-free mice (12). This study raises the possibility that gut microbiota may play a role in MG development. Nevertheless, the existing evidence from conventional epidemiological studies needs to be more adequate in addressing the complexities arising from biases, reverse causality, small sample sizes, and ethical concerns that restrict experimental research. Furthermore, observational studies may not account for potential confounding factors such as comorbidities, medications, and microbiomes at various anatomical sites (13). Consequently, the causal link of the gastrointestinal microbiome with the development of MG remains unexplored. The disentanglement of this causality holds substantial clinical significance, as it has the potential to bridge the existing gaps between classic epidemiological research and emerging novel biomarkers. By establishing the particular association between gut microbiota and MG, valuable insights can be gained, leading to innovative diagnostic and treatment options.

The causal relationship between an outcome and an exposure is investigated by Mendelian randomization (MR) (14), in which single nucleotide polymorphisms (SNPs) are regarded as instrumental variables (IVs) to assess the causality (15). SNPs follow the notion that genetic variants are distributed randomly during meiosis; hence, the possible consequences of reverse causation and the influence of confounding variables are removed. The causal relationship between autoimmune conditions, such as rheumatoid arthritis (16), systemic lupus erythematosus (17), and inflammatory bowel disease (18) with gastrointestinal microbiome has also been investigated. The present research used a two-sample MR approach to examine a possible causal relationship between gastrointestinal microbiota and MG using a genome-wide association study (GWAS) dataset.

## 2 Materials and methods

### 2.1 Study design

**Figure 1:**
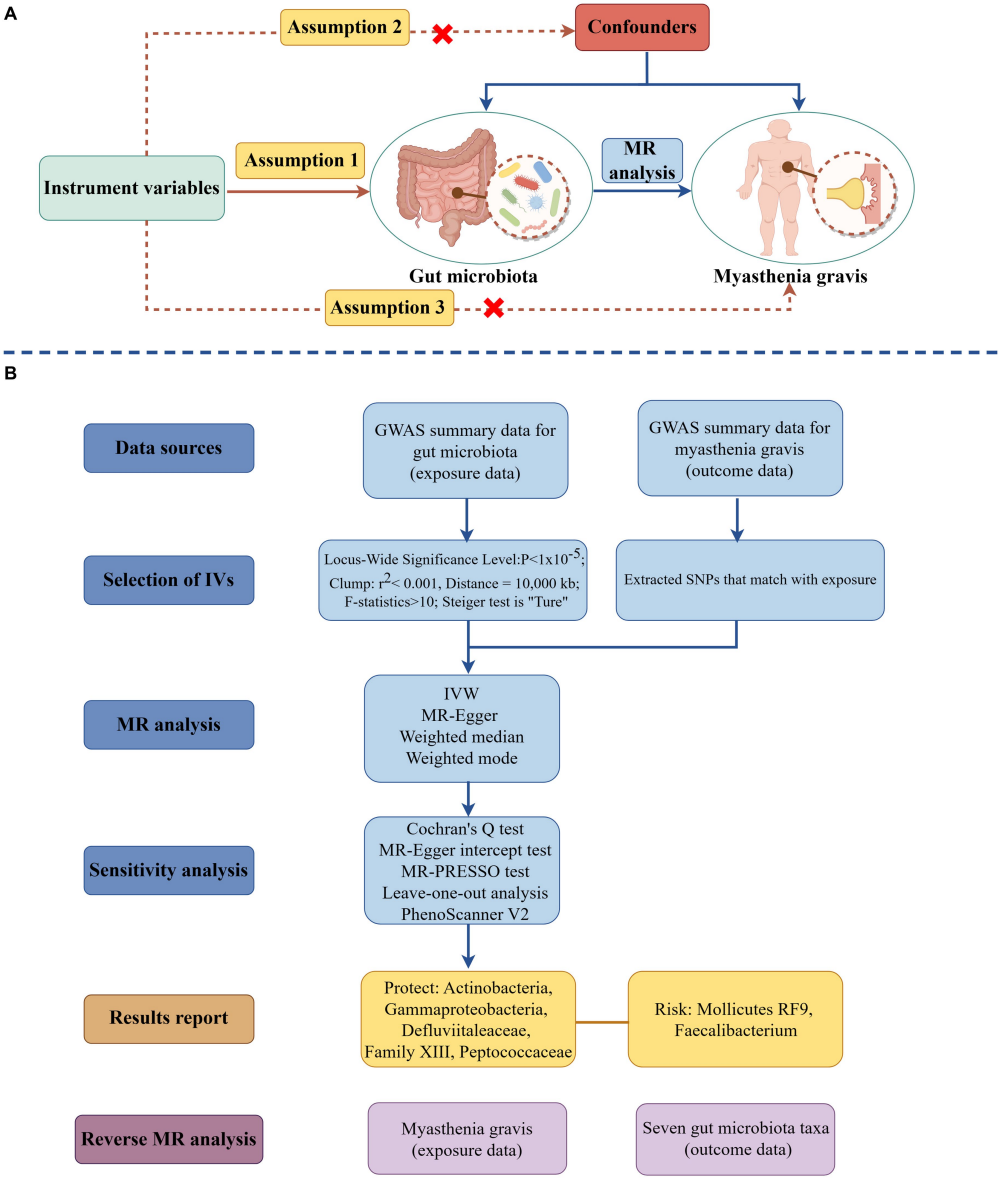
The schematic illustration of the causal relationship between gut microbiota and MG through MR analyses (drawing by Figdraw). **(A)** Principles of Mendelian Randomization; **(B)** The flowchart of the MR analysis. (MR, Mendelian randomization; SNPs, Single nucleotide polymorphisms; IVs, Instrument variables; MG, Myasthenia gravis; and IVW, Inverse variance weighted).

The researchers employed a two-sample MR methodology to examine the causal relationship between the microbiota of the gastrointestinal system and MG. The research design is depicted in Figure 1, which was generated using the Figdraw website.^1^ MR investigations must fulfill the three primary assumptions outlined below: (i) The IVs exhibited a correlation with the gut microbiota. (ii) The IVs demonstrated no significant relationship with any confounding factors. (iii) The IVs only influenced the MG via the gut microbiota pathway (19). The STROBE-MR guideline guided the study design (20), and the checklist may be found in the Supplementary material.

### 2.2 Ethics statement

The original studies were conducted with approval from relevant ethics committees, and all enrolled participants provided informed consent. As this study utilized only publicly available summary-level data from these published studies, additional ethical approval was not required.

### 2.3 Data source

The most extensive genome-wide association study (GWAS) on gastrointestinal microbiota was performed by the utilization of the MiBioGen consortium, which brought together 16S rRNA gene sequencing profiles from 24 cohorts (18,340 individuals) (21). The genetic data on gut microbiota from this were utilized in our current study as exposure. The analysis of microbiome quantitative trait loci (mbQTL) mapping was conducted exclusively on taxa that had been identified in a minimum of 10% of the samples. Here, 211 taxa, encompassing nine phyla, 16 classes, 20 orders, 35 families, and 131 genera, were shown. The original research work provided significant information and details on the microbiota data.

The most comprehensive meta-GWAS conducted in Italy and the United States was utilized for the meta-GWAS data for MG, which included 1,873 patients and 36,370 controls matched for age and sex (22). The study exclusively recruited individuals diagnosed with MG who tested positive for antiacetylcholine receptor antibodies (AChR^+^).

### 2.4 Instrument variables selection

To ensure the authenticity and accuracy of conclusions regarding the causal link between the gut microbiota and MG risk, optimal IVs were selected through the following quality control steps. Firstly, the number of SNPs under the genome-wide significance threshold (*p* < 5 × 10^−8^) was limited, potentially leading to false-negative results due to insufficient statistical power. Therefore, to identify more potential causal associations, the locus-wide significance threshold (*p* < 1 × 10^−5^) was employed for selecting SNPs associated with exposure. This approach, used in the original study (21), has been widely adopted in previous MR studies (23, 24). Secondly, to adhere to MR principles, we ensured no linkage disequilibrium (LD) among the included IVs, as strong LD could lead to biased results (25). In this study, LD analysis was performed using European-based 1000 Genome Project (26) data, setting a threshold of *R^2^* < 0.001 and a clumping distance of 10,000 kb. Thirdly, an essential step in MR involved ensuring that the effects of single SNPs on the exposure corresponded to the same allele as their effects on the outcome (25). To adhere to this principle, palindromic SNPs with intermediate allele frequencies and inconsistent allele representations between exposure and outcome samples were excluded. Fourthly, the *F*-statistic, indicative of the strength of the association between genetic variants and exposure (27), was calculated for each SNP (beta^2^/se^2^) (28, 29), with those showing less statistical power (*F*-statistics < 10) being removed (30). Finally, the Steiger test was applied to each SNP to determine if the *R^2^* for the exposure surpassed that of the outcome, thereby excluding SNPs where the test indicated a “FALSE” directional effect.

### 2.5 Statistical analysis

This study employed four methods to investigate the causal link between gut microbiota and MG: inverse variance weighted (IVW) (31), MR-Egger regression (32), weighted median (33), and weighted mode (34). The IVW method, which compiles Wald ratio estimates of each instrumental SNP via a meta-analysis-like model, provides an unbiased overall effect estimate if no horizontal pleiotropy is present (35). Consequently, IVW was prioritized as the primary method. Additionally, IVW results were corroborated and validated using MR-Egger regression, weighted median, and weighted mode. MR-Egger regression, operating under the Instrument Strength Independent of Direct Effect (InSIDE) assumption, considers all genetic variants as potentially invalid IVs and includes an intercept for estimating average pleiotropic effects (32). A zero intercept in MR-Egger regression suggests the absence of horizontal pleiotropy, aligning its results with IVW. The weighted median approach can yield a consistent estimate even if up to 50% of the IVs are invalid, though with reduced statistical power (33). Results from the weighted median were prioritized when the MR-PRESSO global test indicated pleiotropy or the MR-Egger intercept’s *p* value <0.05. The weighted mode approach remains applicable even when the remaining IVs do not satisfy the prerequisites for causal inference in the MR method, as long as most of the IVs have consistent causal estimations (34). The Benjamini and Hochberg procedure (BH procedure) is used to test multiple independent hypotheses simultaneously and is designed to control for false discovery rate (FDR) (36). To account for multiple exposures, the statistical significance of the MR effect estimate was defined as the FDR *p* value <0.1. Regarding the implications of the study findings, it was determined that a significant causal association between exposure and outcome was deemed to be present when the IVW *p* value <0.05 and the the FDR *p* value <0.1; meanwhile, the IVW *p* value <0.05 but the FDR *p* value ≥0.1 was considered as a suggestive association. This method has also been used in previous MR studies (23, 37).

### 2.6 Sensitivity analysis

The reliability and stability of the findings were evaluated by conducting a series of critical sensitivity assessments. The Cochran Q statistic in IVW was used to evaluate the heterogeneity in effect sizes produced by the selected genetic IVs (31). The lack of heterogeneity was indicated by a *p* value greater than 0.05. Subsequently, the MR-Pleiotropy Residual Sum and Outlier (MR-PRESSO) method and the MR-Egger intercept were utilized to assess the presence of horizontal pleiotropy (32, 38). In the event of substantial horizontal pleiotropy being present in the MR-PRESSO global test, the remaining SNPs would be recalculated using the IVW method, and the outliers would be excluded. The potential pleiotropy of the SNP was evaluated using the MR-Egger regression intercept; a *p* value of more than 0.05 denotes the lack of horizontal pleiotropy. Additionally, to mitigate the potential effects of horizontal pleiotropy resulting from a single SNP, a leave-one-out analysis was conducted, wherein each SNP was carefully excluded one at a time. Finally, a reanalysis was conducted, wherein SNPs linked to possible confounding factors were excluded and were retrieved from the PhenoScanner V2 database (39). These included age (40), asthma (41), and sedentary behavior (42). The analyses were conducted using the following software packages of R Software 4.3.0 with their versions: “TwoSampleMR” (version 0.5.7), “MendelianRandomization” (version 0.8.0), and “MRPRESSO” (version 1.0).

### 2.7 Reverse Mendelian randomization

Myasthenia gravis was considered the exposure variable to investigate the possibility of reverse causality between MG and the screened taxa of the gastrointestinal microbiota. In contrast, gut microbiota taxa were identified as the outcome variable. The SNPs associated with MG as IVs were extracted, and a reverse MR investigation was undertaken.

## 3 Results

### 3.1 Selection of instrumental variables

Following a rigorous sequence of quality control procedures, a total of 99, 172, 212, 349, and 1,175 SNPs associated with gut microbiota at the phylum, class, order, family, and genus levels were identified. The *F*-statistic calculated for the IVs exceeded a value of 10, hence showing the absence of any weak instrument bias. The specific IVs utilized in the study are shown in Supplementary Table S1.

### 3.2 Causal impacts of gastrointestinal microbiota on MG

**Figure 2:**
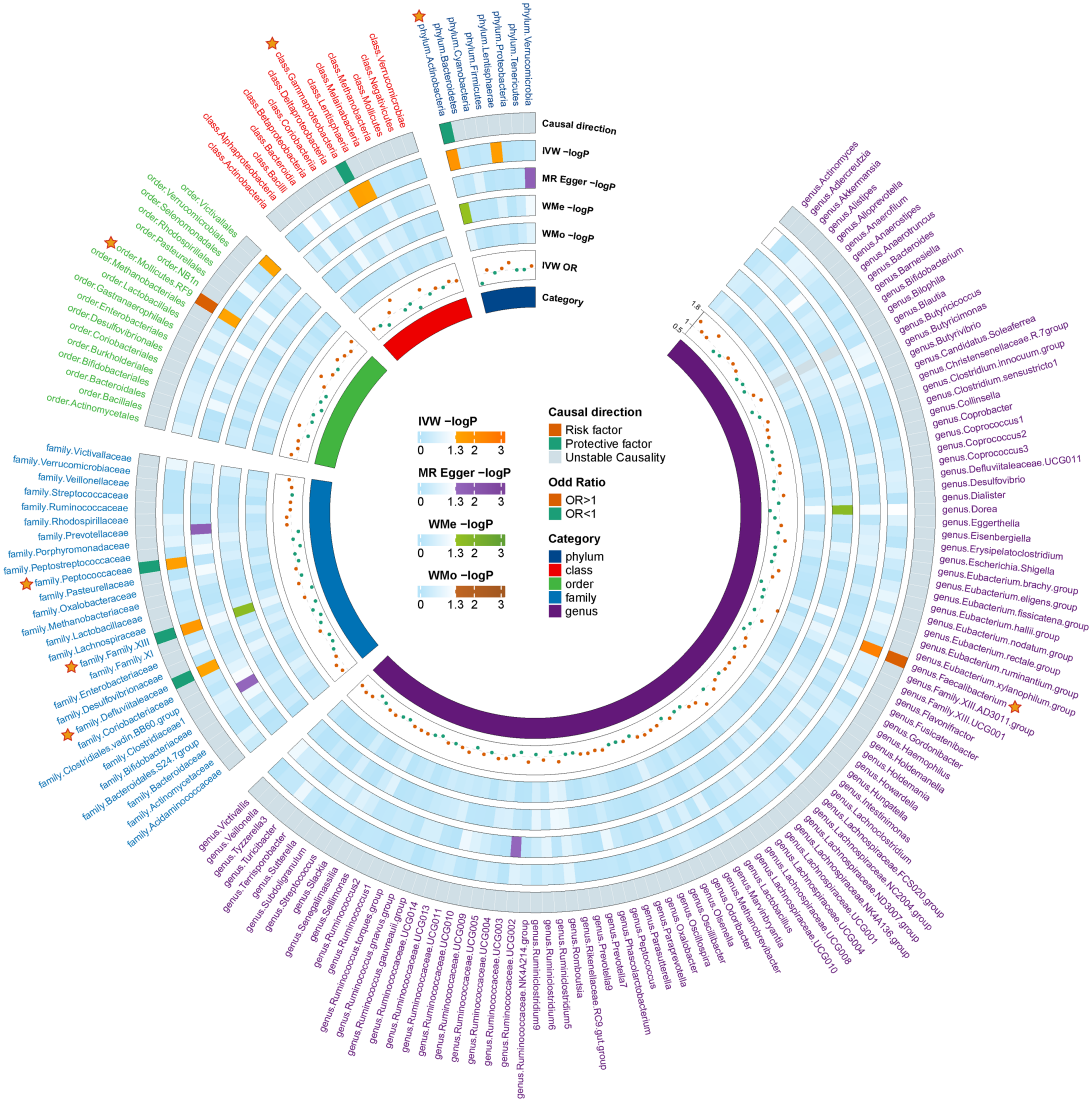
Preliminary MR estimates for the associations between gut microbiota and the risk of MG. The circle from the outer to the inner represented the causal direction, IVW, MR-Egger, weighted median, weighted mod, IVW-or, and gut microbiota category, respectively. The outermost circle is derived from the IVW *p* value <0.05, while the OR directions of the four MR methods are consistent. MG, Myasthenia gravis; IVW, Inverse variance weighted; and OR, Odds ratio.

**Figure 3:**
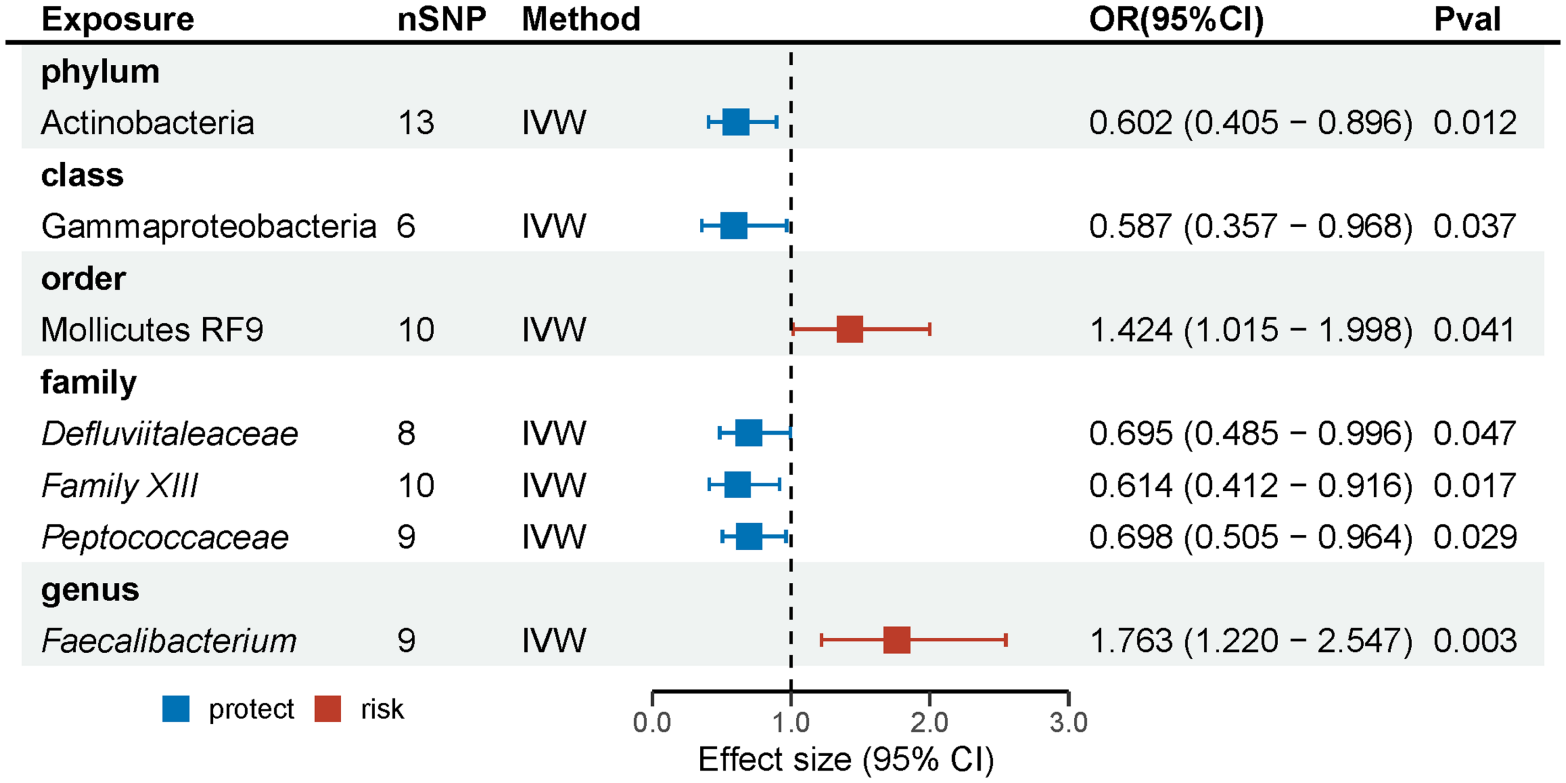
Causal effects of gut microbiota on MG. (OR, odds ratio; CI, confidence interval).

Following the initial MR analysis, the outcomes pertaining to the investigations were acquired on the link between genetically proxied gut microbiota taxa and the risks associated with MG. These findings have been shown in Figure 2; Supplementary Table S2. Seven suggestive causal associations between the gastrointestinal microbiota and MG were identified based on the outcomes of the primary MR analysis, as seen in Figure 3.

Analysis revealed that several microbial taxa have suggestive causal associations with MG. Specifically, the phylum Actinobacteria (OR: 0.602, 95% CI: 0.405–0.896, *p* = 0.012), class Gammaproteobacteria (OR: 0.587, 95% CI: 0.357–0.968, *p* = 0.037), families *Defluviitaleaceae* (OR: 0.695, 95% CI: 0.485–0.996, *p* = 0.047), *Family XIII* (IVW: OR: 0.614, 95% CI: 0.412–0.916, *p* = 0.017; weighted median: OR: 0.538, 95% CI: 0.320–0.905, *p* = 0.020), and *Peptococcaceae* (OR: 0.698, 95% CI: 0.505–0.964, *p* = 0.029) had suggestive protective effects on MG, while order Mollicutes RF9 (OR: 1.424, 95% CI: 1.015–1.998, *p* = 0.041) and genus *Faecalibacterium* (OR: 1.763, 95% CI: 1.220–2.547, *p* = 0.003) were suggestive risk factors for MG. Although consistent, these results did not achieve significance with other methods and did not pass the FDR correction; thus, they are considered as suggestive causal association. Comprehensive outcomes for these seven taxa are detailed in Table 1.

**Table 1:** MR analysis for the casual association between gut microbiota and MG.

Exposure	Method	nSNP	OR	95% CI	*p* value	FDR *p* value
Phylum						
Actinobacteria	IVW	13	0.602	0.405–0.896	**0.012**	0.112
	MR-Egger	13	0.533	0.103–2.744	0.467	0.925
	Weighted median	13	0.586	0.341–1.007	0.053	0.408
	Weighted mode	13	0.523	0.210–1.306	0.190	0.856
Class						
Gammaproteobacteria	IVW	6	0.587	0.357–0.968	**0.037**	0.244
	MR-Egger	6	0.774	0.169–3.539	0.758	0.952
	Weighted median	6	0.617	0.324–1.174	0.141	0.908
	Weighted mode	6	0.613	0.266–1.415	0.305	0.933
Order						
Mollicutes RF9	IVW	10	1.424	1.015–1.998	**0.041**	0.443
	MR-Egger	10	1.679	0.654–4.306	0.313	0.781
	Weighted median	10	1.372	0.856–2.198	0.189	0.907
	Weighted mode	10	1.298	0.674–2.502	0.456	0.898
Family						
*Family XIII*	IVW	10	0.614	0.412–0.916	**0.017**	0.455
	MR-Egger	10	0.308	0.087–1.092	0.106	0.410
	Weighted median	10	0.538	0.320–0.905	**0.020**	0.785
	Weighted mode	10	0.542	0.264–1.113	0.130	0.944
*Defluviitaleaceae*	IVW	8	0.695	0.485–0.996	**0.047**	0.489
	MR-Egger	8	0.463	0.102–2.105	0.356	0.735
	Weighted median	8	0.720	0.456–1.136	0.158	0.858
	Weighted mode	8	0.698	0.362–1.345	0.318	0.944
*Peptococcaceae*	IVW	9	0.698	0.505–0.964	**0.029**	0.455
	MR-Egger	9	0.889	0.414–1.908	0.771	0.980
	Weighted median	9	0.774	0.494–1.211	0.262	0.858
	Weighted mode	9	0.930	0.516–1.677	0.816	0.944
Genus						
*Faecalibacterium*	IVW	9	1.763	1.220–2.547	**0.003**	0.299
	MR-Egger	9	1.462	0.715–2.988	0.332	0.903
	Weighted median	9	1.469	0.888–2.431	0.134	0.997
	Weighted mode	9	1.413	0.771–2.590	0.296	0.994

### 3.3 Sensitivity analysis

The sensitivity of MR analysis concerning the seven gut microbiota species linked to MG was assessed to mitigate potential bias. According to the Cochrane’s *Q* test results, there was no statistically significant heterogeneity among the chosen SNPs (*p* > 0.05). Based on the outcomes of the MR-Egger intercept test and MR-PRESSO test for pleiotropy, the MR investigation did not exhibit pleiotropy (*p* > 0.05). Table 2 presents the findings of the pleiotropy and heterogeneity analyses. Therefore, this study’s IVW results should be considered the primary outcome because, without horizontal pleiotropy, the IVW results would be unbiased (35). The risk estimations for genetically predicted in leave-one-out analyses did not exhibit any substantial changes, indicating that individual SNPs did not influence the causal relationship (Supplementary Figure S1). No indication of horizontal pleiotropy was observed in our analysis, which is evidenced by the absence of any significant findings in the scatter and forest plots (Supplementary Figures S2, S3).

**Table 2:** Pleiotropy and heterogeneity analysis for IVs of seven gut microbiota taxa associated with MG.

Exposure	Pleiotropy				Heterogeneity	
	MR-Egger intercept test		MR-PRESSO global test		Cochran’s *Q* tests (IVW)	
	Egger-intercept	*p* value	RSS obs	*p* value	*Q*	*Q*_*p* value
Phylum						
Actinobacteria	0.008	0.883	10.075	0.755	8.423	0.748
Class						
Gammaproteobacteria	−0.022	0.726	2.403	0.902	1.721	0.886
Order						
Mollicutes RF9	−0.015	0.723	8.060	0.707	6.617	0.677
Family						
*Family XIII*	0.051	0.293	4.385	0.941	3.587	0.936
*Defluviitaleaceae*	0.052	0.607	10.968	0.336	8.502	0.290
*Peptococcaceae*	−0.026	0.516	7.386	0.697	5.327	0.722
Genus						
*Faecalibacterium*	0.023	0.568	7.745	0.672	6.119	0.634

Given that all SNPs’ *F*-statistics were greater than 10, there was no bias due to weak IVs (Supplementary Table S1). Notably, the PhenoScanner V2 revealed that rs9725395 correlated with age, rs9536330 was associated with asthma, and rs638542 was linked with sedentary behavior. Excluding these SNPs from the IVs did not alter the nominal significance of the observed causal relationships between gut microbiota and MG. Insignificant heterogeneity and pleiotropy were also observed.

### 3.4 Reverse MR

Reverse MR analysis was performed on seven gastrointestinal microbiota taxa identified during the discovery phase. According to the findings, there were no reverse causal relationships between MG and gastrointestinal microbiota. Moreover, sensitivity analyses revealed no directional pleiotropy, heterogeneity, or outliers for the causal effect of MG on particular gastrointestinal microbiota. Supplementary Table S3 displays the complete outcomes of the reverse MR analysis.

## 4 Discussion

This study employed a two-sample MR analysis to explore the causal impact of gastrointestinal microbiota on MG. Results indicate a suggestive causal relationship between specific microbiota and MG. Notably, the phylum Actinobacteria, class Gammaproteobacteria, and the families *Defluviitaleaceae*, *Family XIII*, and *Peptococcaceae* appeared to have protective roles against MG. In contrast, the order Mollicutes RF9 and the genus *Faecalibacterium* emerged as potential risk factors. Furthermore, the analysis found no reverse causal relationships between MG and these seven microbiota taxa. These insights could be instrumental in developing novel therapeutic and preventive strategies for MG.

The study of the gut microbiome’s influence on immunity has attracted significant attention in recent years (43). A growing body of research indicates a strong association between dysbiosis of the gastrointestinal microbiome and the progression of MG (44, 45). The primary emphasis of the comparative study conducted on the MG and healthy control group pertained to the diversity and abundance of the gut microbiota, as well as examining the metabolites produced by the gut microbiota. High alpha-phylogenetic diversity, typically indicative of good health (46), was found to be reduced in MG patients compared to healthy controls (9, 12), although some studies report no significant difference in alpha-phylogenetic diversity between these groups (10, 47). This discrepancy highlights the need for further research to establish a definitive link between gut microbiota diversity and MG. Previous studies have shown that the abundance of the gut microbiota differed between the MG group and the healthy control group. For instance, a case–control study demonstrated that the MG group exhibited reduced levels of the genera *Clostridium* and *Eubacterium* and species *F. prausnitzii*. In contrast, higher levels of phyla Proteobacteria and Bacteroidetes and genera *Streptococcus* and *Parasutterella* were observed in the MG group (9). The insufficiency of FOXP3^+^ CD4+ regulatory T cells (T_reg_ cells) is central to comprehending intestinal microbiota dysbiosis’s role in MG’s pathogenesis. T_reg_ cells are a subpopulation of CD4^+^ T cells that express both the CD25 and FOXP3 transcription factors. These cells are crucial in preserving immunological tolerance and immune homeostasis (48). The alterations in the relative proportions of bacterial taxa within the MG group can influence the differentiation of FOXP3^+^ CD4^+^ T_reg_ cells. As an example, the genus *Clostridium* has the potential to enhance the production of 2,3-dioxygenase and TGF-β1, hence potentially facilitating the differentiation of naive CD4^+^ T cells into FOXP3^+^ CD4^+^ T_reg_ cells (49). In contrast, there was a significant decrease in the abundance of the genus *Clostridium* within the MG group in comparison to the healthy control (9). This leads to insufficient frequency of FOXP3^+^ CD4^+^ T_reg_ cells, thereby weakening their ability to inhibit the production of autoantibodies. Short-chain fatty acids (SCFAs), non-nutritional substances produced by gut microbiota, play significant roles in physiological regulation. Dysbiosis in the gut microbiota can alter SCFA production, particularly propionate and butyrate, known for their immunoregulatory functions, including the augmentation of FOXP3^+^ CD4^+^ T_reg_ cells (50). A study indicated that SCFA levels in MG patients were lower compared to healthy controls (9). Therefore, the gut microbiota may enhance the population of FOXP3^+^ CD4^+^ T_reg_ cells through increased SCFA production, thereby potentially exerting a protective effect against MG.

Early diagnosis of MG, which is crucial for effective treatment, may benefit from identifying microbial biomarkers, as recent studies suggest a link between gut microbiota and MG (5). Zheng et al. reported that MG subjects typically showed reduced operational taxonomic units (OTUs) associated with the *Lachnospiraceae* and *Ruminococcaceae* families (12). Additionally, animal studies have demonstrated that a probiotics mixture (IRT5)—comprising *Streptococcus thermophilus, Lactobacillus reuter, Bifidobacterium bifidium, Lactobacillus acidophilus*, and *Lactobacillus casei*—can mitigate pro-inflammatory responses and lower AChR antibody levels in MG rat models by increasing Foxp3^+^CD4^+^T_reg_ cells (51). However, our study did not replicate these findings, possibly due to the limited sample size. It was observed that the phylum Actinobacteria, class Gammaproteobacteria, and families *Defluviitaleaceae*, *Family XIII*, and *Peptococcaceae* may have protective effects against MG. Actinobacteria is a diverse group of Gram-positive bacteria (52), with several studies demonstrating potential advantages for human health. Research of Moris et al. (10) showed that phylum Actinobacteria were less abundant in patients with MG than in the healthy control group. This research is consistent with our MR analysis. However, the exact mechanism of the protective effect of phylum Actinobacteria against MG is currently unknown. In addition, it is worth noting that the current body of research on the correlation between the other four gut microbiota taxa and MG is limited. Therefore, it is imperative to use caution when interpreting the findings, and further investigation is required to elucidate their potential significance in the context of MG. Future research should investigate the biological mechanisms linking these gut microbiota taxa with MG, potentially offering novel strategies for its prevention and treatment.

In addition, this MR analysis identified the order Mollicutes RF9 and the genus *Faecalibacterium,* a Gram-positive bacterium notably prevalent in the gut microbiome of healthy adults, as potential risk factors for MG. Different findings emerged from a case–control study in China, which found that *Faecalibacterium* was significantly less abundant in MG patients than in healthy controls (53). Conversely, another study reported a higher abundance of *Faecalibacterium* in MG patients relative to those with non-inflammatory neurological conditions (54). *Faecalibacterium* is typically regarded as beneficial, contributing to immune regulation, gut barrier integrity, and microbiota balance (55, 56). However, some MR studies have identified *Faecalibacterium* as a risk factor in diseases like female infertility and age-related macular degeneration, with the underlying causes for these discrepancies remaining unclear (57, 58). Future clinical and experimental research is crucial to elucidate the mechanisms linking *Faecalibacterium* with MG. Additionally, larger and more diverse gut microbiota GWAS datasets are needed to enhance the robustness and objectivity of MR study findings. Notably, Mollicutes, a class of bacteria characterized by the absence of a cell wall and a small genome, are also of interest in this context (59). Studies have shown that changes in microbial composition enable microbes and their metabolites to invade and pass through the gut barrier, evade immune intervention, and enter the circulation, leading to immune activation and chronic systemic inflammation (45). An MR study demonstrated that as Mollicutes lack a cell wall, they may be more invasive and capable of crossing the gut barrier (59). Based on their structural properties, it is hypothesized that Mollicutes, particularly the order Mollicutes RF9, might invade more easily across the intestinal barrier, potentially contributing to MG development. However, due to the lack of direct research studies on the correlation between order Mollicutes RF9 and MG, caution must be exercised in interpreting the findings.

Su et al. (60) also employed MR to explore the causal link between gut microbiota and MG. Their study, using MG-related GWAS data from the FinnGen R9 database, could not differentiate the effects of gut microbiota on various antibody types of MG. In contrast, this study focused exclusively on AChR antibody-positive MG patients; hence, it could not establish causal relationships for other antibody-positive MG types. The differing datasets led to varying results between this study and Su et al.’s, suggesting that gut microbiota may influence different MG types differently. Both studies identified a suggestive causal relationship between gut microbiota and MG, with variations in specific gut microbiota taxa, thereby expanding the scope for future research and strategies to modulate gut microbiota for MG prevention and treatment. Notably, both studies highlighted the potential protective role of the *Defluviitaleaceae* family against MG, suggesting its significance in MG management.

From a clinical perspective, this study’s findings could contribute to developing innovative prevention and treatment strategies for MG. Potential interventions include small-molecule antibiotics, specific probiotic strains, bioactive metabolites, and fecal microbiota transplantation (FMT) (5, 44). However, as this research area is still nascent, further investigations are required to validate these initial results and identify the most effective approaches. Additionally, the inherent complexity and individual variability of gut microbiota necessitate the consideration of personalized treatments.

However, it is vital to recognize the limitations of the present investigation. Initially, it should be noted that the original research on MG exclusively included individuals with AChR antibody-positive MG. Consequently, the absence of other types of MG patients, such as those with MuSK antibody-positive and LRP4 antibody-positive, precluded us from doing additional subgroup analyses to ascertain a more precise impact of the relationship. Secondly, it is noteworthy that the GWAS summary data used in the present analysis predominantly consisted of individuals of European ancestry. Biased estimations may arise due to this, necessitating caution in generalizing our findings to other ethnic groups. In addition, the assessment of gut microbiota taxa was limited to summary statistics due to the absence of individual-level data. To explore possible variations among groups, further analyses pertaining to population stratification, such as gender and age, might be undertaken. Finally, bacteria are the predominant component in the gut microbiome, but viruses, fungi, and archaea are also present. The causal relationship between other gut microbes and MG could not be explored due to the lack of relevant GWAS data.

## 5 Conclusion

In conclusion, this MR study supports a suggestive causal relationship between gut microbiota and MG, offering valuable insights for novel therapeutic and preventive strategies. Future randomized controlled trials are essential to fully understand the role and therapeutic implications of gut microbiota in MG’s pathogenesis. At the same time, this study suffers from limitations such as using only European ancestry and AChR antibody-positive GWAS data, which may diminish the general applicability of our findings. We look forward to more including larger sample size and more ethnic group GWAS data, as well as more individual level GWAS data to meet the requirement of obtaining more comprehensive and reliable findings on the causal relationship between gut microbiota and MG.

## Data availability statement

The original contributions presented in the study are included in the article/Supplementary material, further inquiries can be directed to the corresponding author.

## Ethics statement

Our analyses used published studies or publicly available GWAS abstract data. All studies were conducted with the approval of the appropriate institutional ethics committees, and therefore did not require additional ethical approval.

## Author contributions

CM: Writing – original draft. AH: Writing – original draft. ZW: Writing – original draft. XQ: Writing – review & editing. JT: Writing – review & editing.

## Footnotes

^1^www.figdraw.com
