# Supplementary material for: Causal relationship between gut microbiota and myasthenia gravis: a two-sample Mendelian randomization study
Source: Front Neurol. 2024 Jan 25;15:1309530. doi: 10.3389/fneur.2024.1309530 (PMC10850378; doi:10.3389/fneur.2024.1309530)
Supplement: Supplementary file 1 [file Data_Sheet_1.docx]

**Supplementary Figures**

**Supplemental Figure S1.** The leave-one-out sensitivity analysis for the association between gut microbiota and MG.

**Supplemental Figure S2.** The scatter plots of the 4 MR approaches for association between gut microbiota and MG.

**Supplemental Figure S3.** The forest plots for the association between gut microbiota and MG.

**Supplemental Figure S1.** The leave-one-out sensitivity analysis for the association between gut microbiota and MG.

1. The leave-one-out sensitivity analysis for the association between phylum Actinobacteria and MG


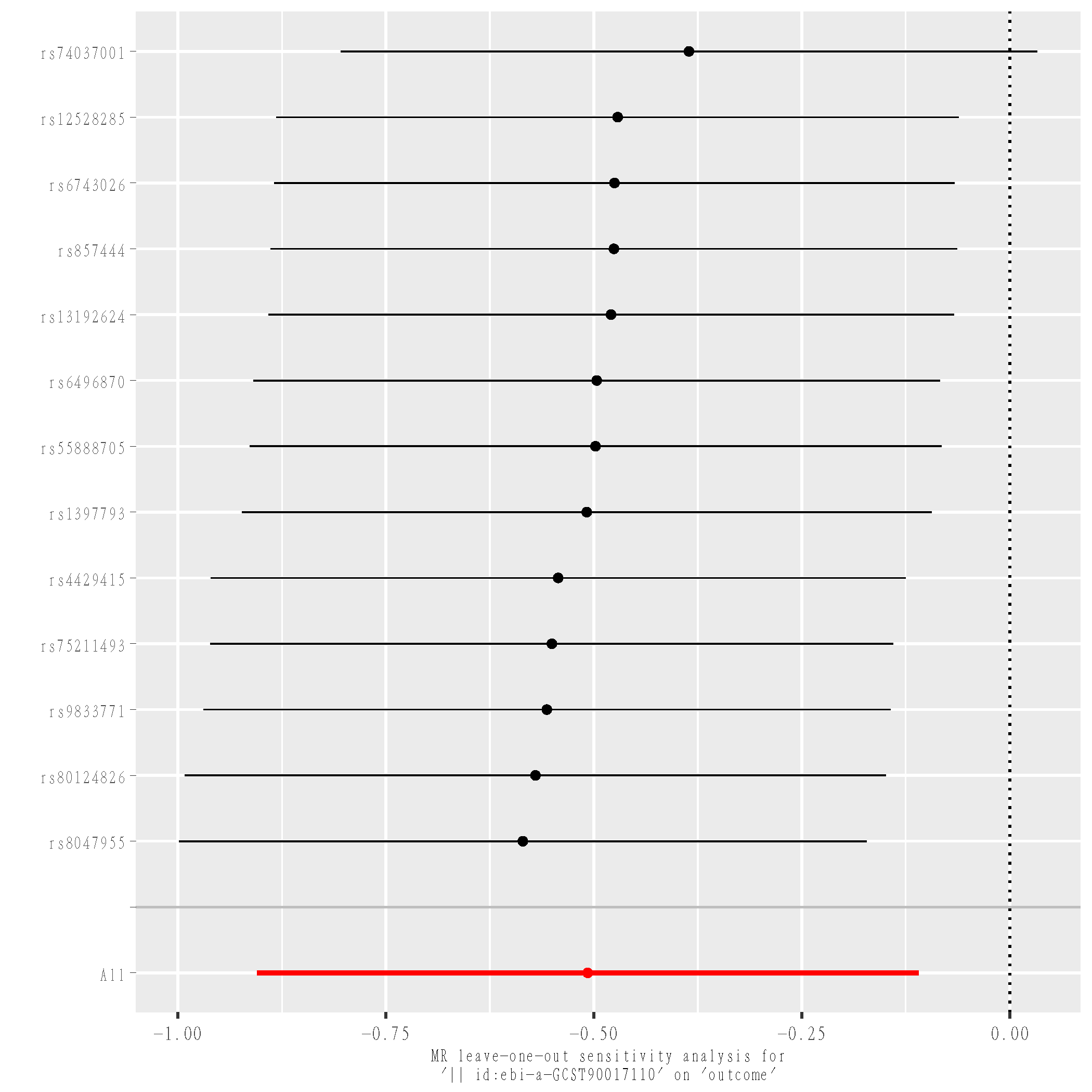


2. The leave-one-out sensitivity analysis for the association between class Gammaproteobacteria and MG


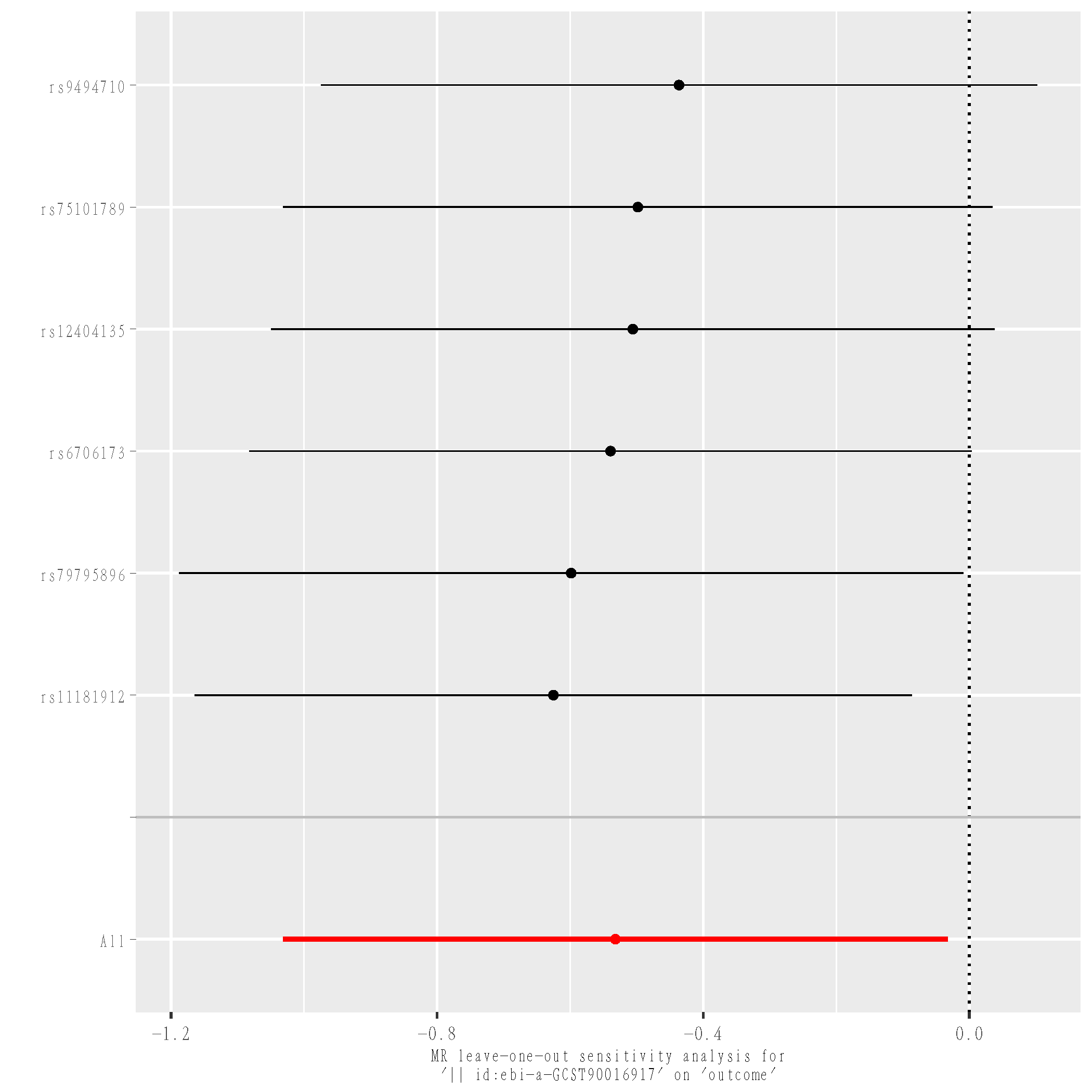


3. The leave-one-out sensitivity analysis for the association between order Mollicutes RF9 and MG.


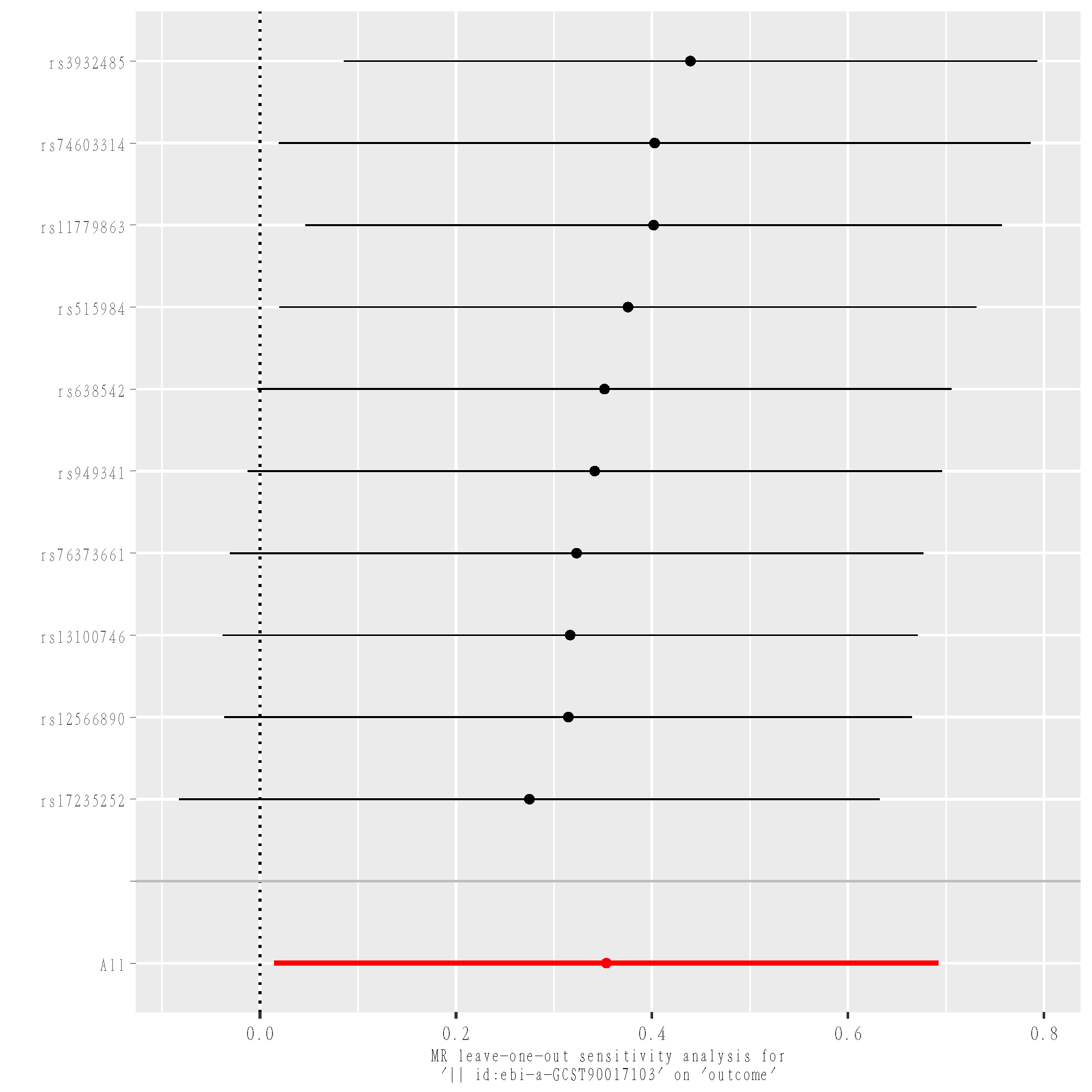


4. The leave-one-out sensitivity analysis for the association between family *Defluviitaleaceae* and MG.


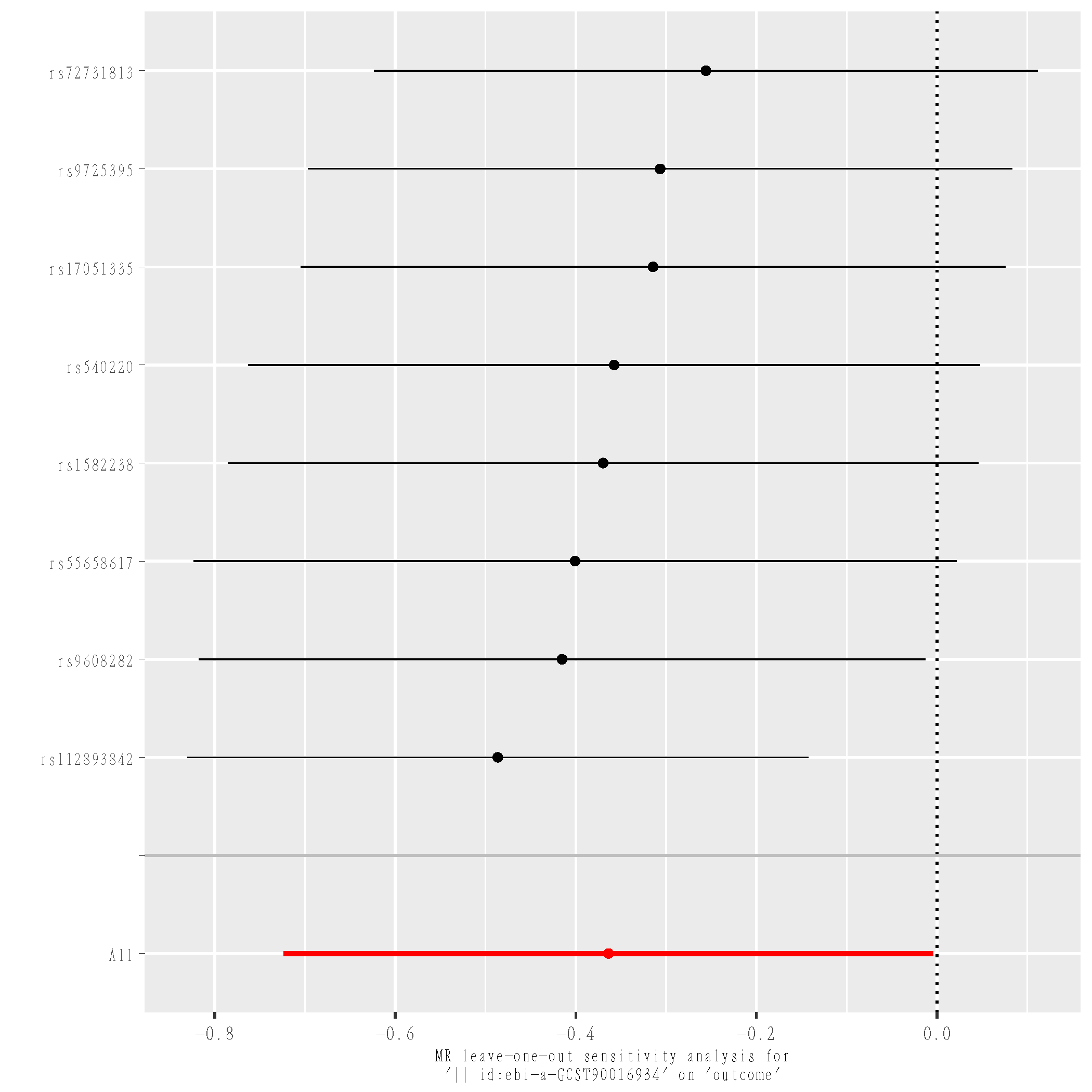


5. The leave-one-out sensitivity analysis for the association between family *Family XIII* and MG.

**
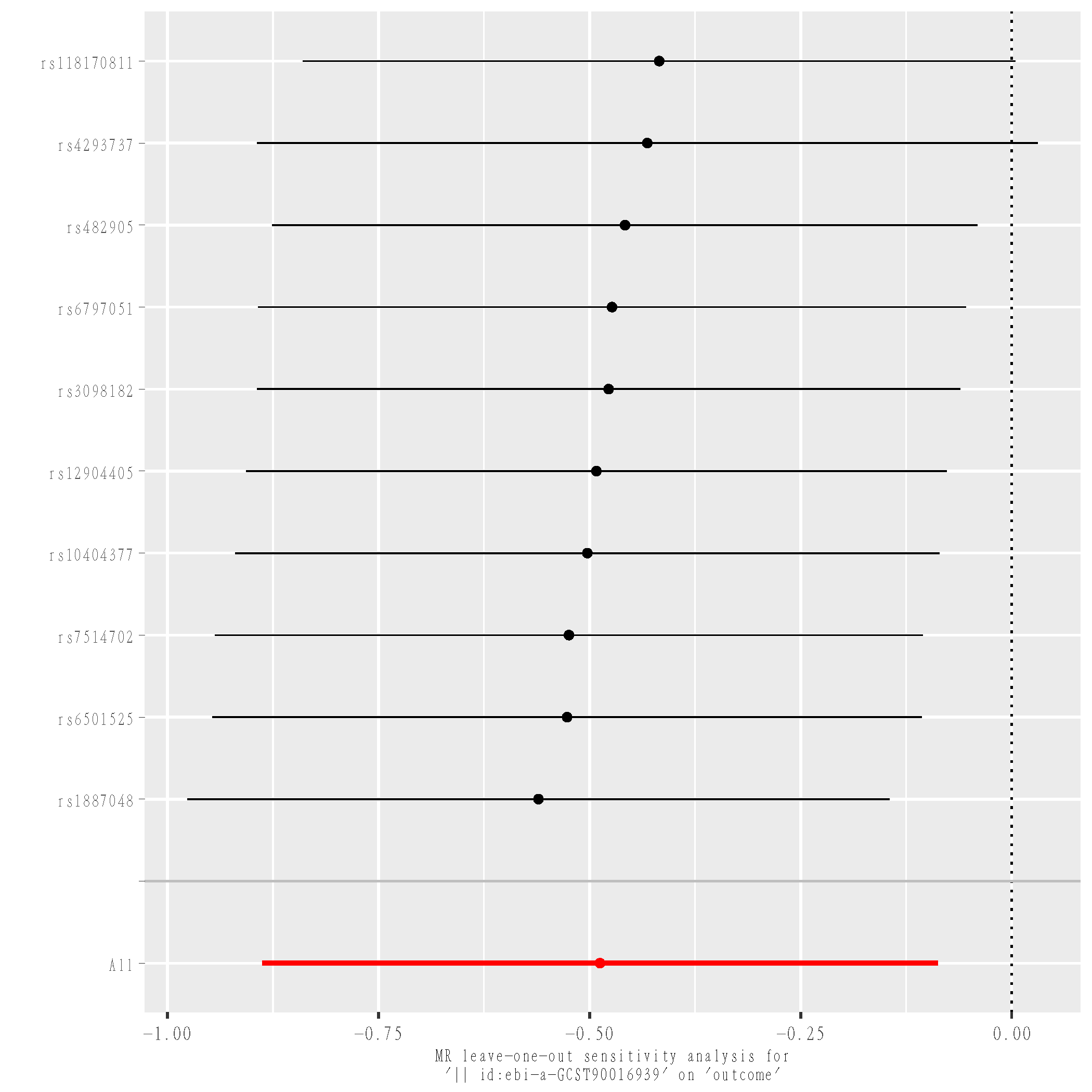
**

6. The leave-one-out sensitivity analysis for the association between family *Peptococcaceae* and MG


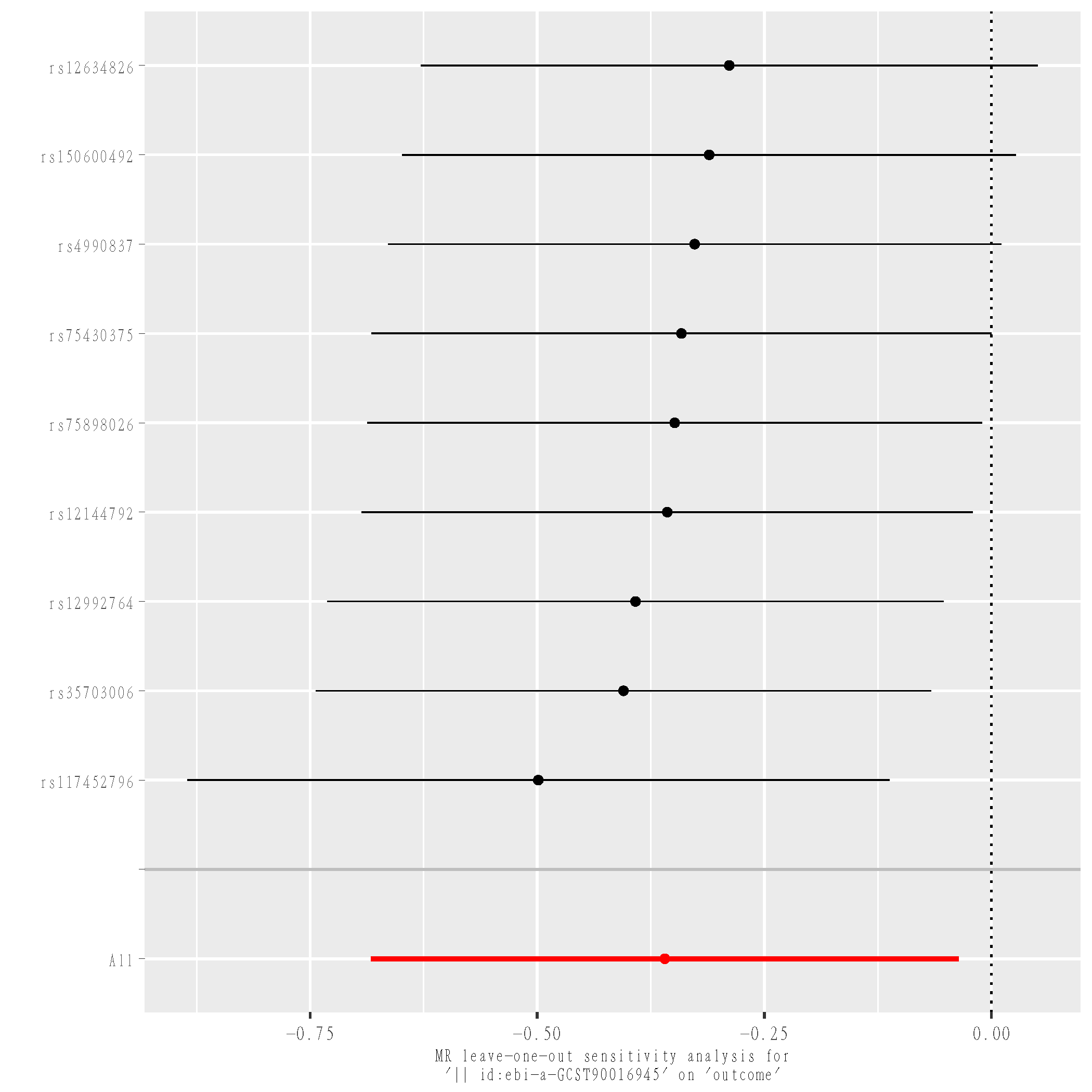


7. The leave-one-out sensitivity analysis for the association between genus *Faecalibacterium* and MG

**
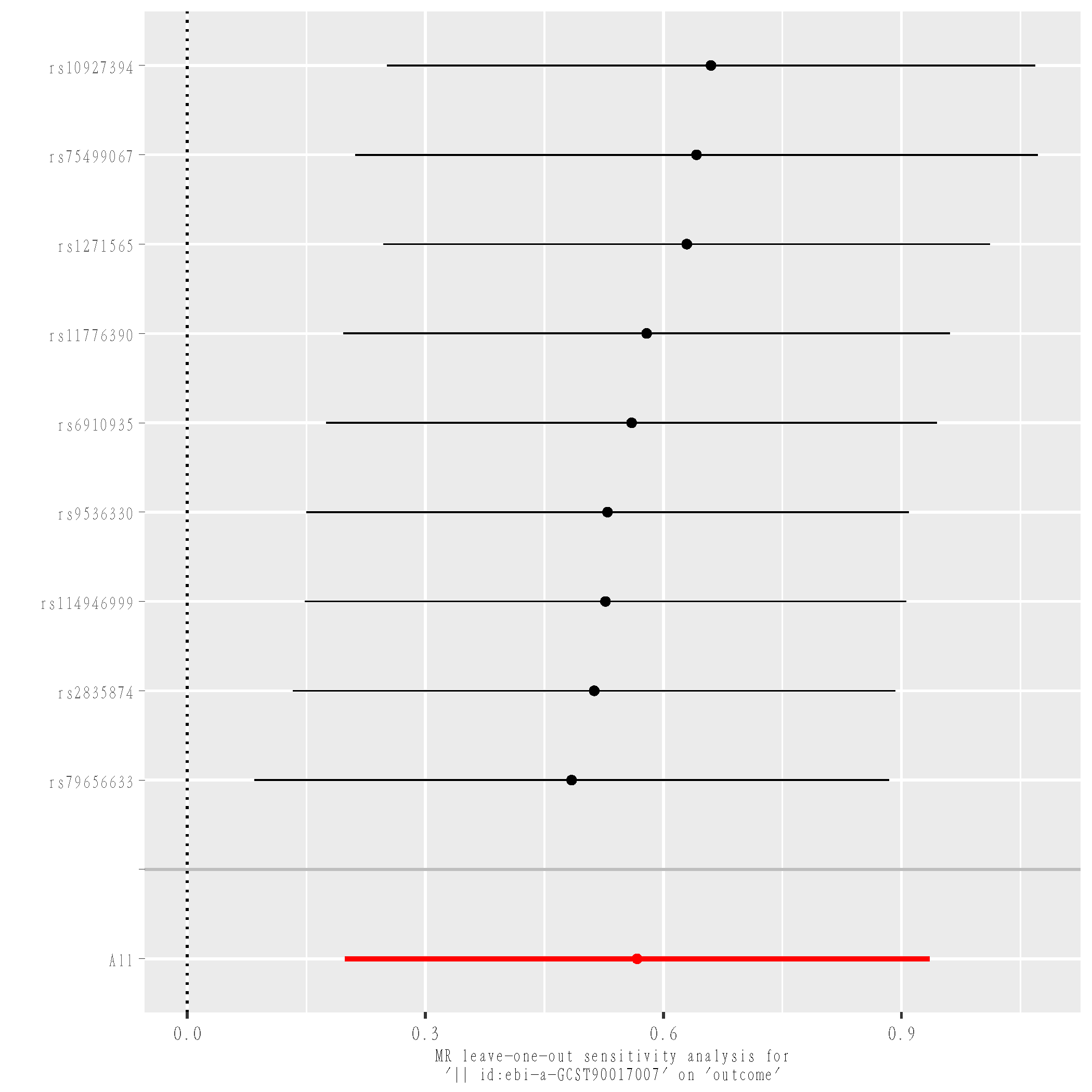
**

Leave-one-out plot helps determine whether the overall effect is altered by one or more specific genetic variants by sequentially re-evaluating causal estimates after discarding one SNP at a time. The four plots reveal no single SNP distorting overall MR estimates when each SNP is removed from principal MR analyses. The black points denote effect estimates of the gut microbiota after discarding a certain SNP, and black lines signify the corresponding 95% CIs of estimates. Red points symbolize overall causal effect estimate of the gut microbiota on MG using a set of SNPs, and red lines indicate the corresponding 95% CIs.

**Supplemental Figure S2.** The scatter plots of the 4 MR approaches for association between gut microbiota and MG.

1. The scatter plots of the 4 MR approaches for association between phylum Actinobacteria and MG.


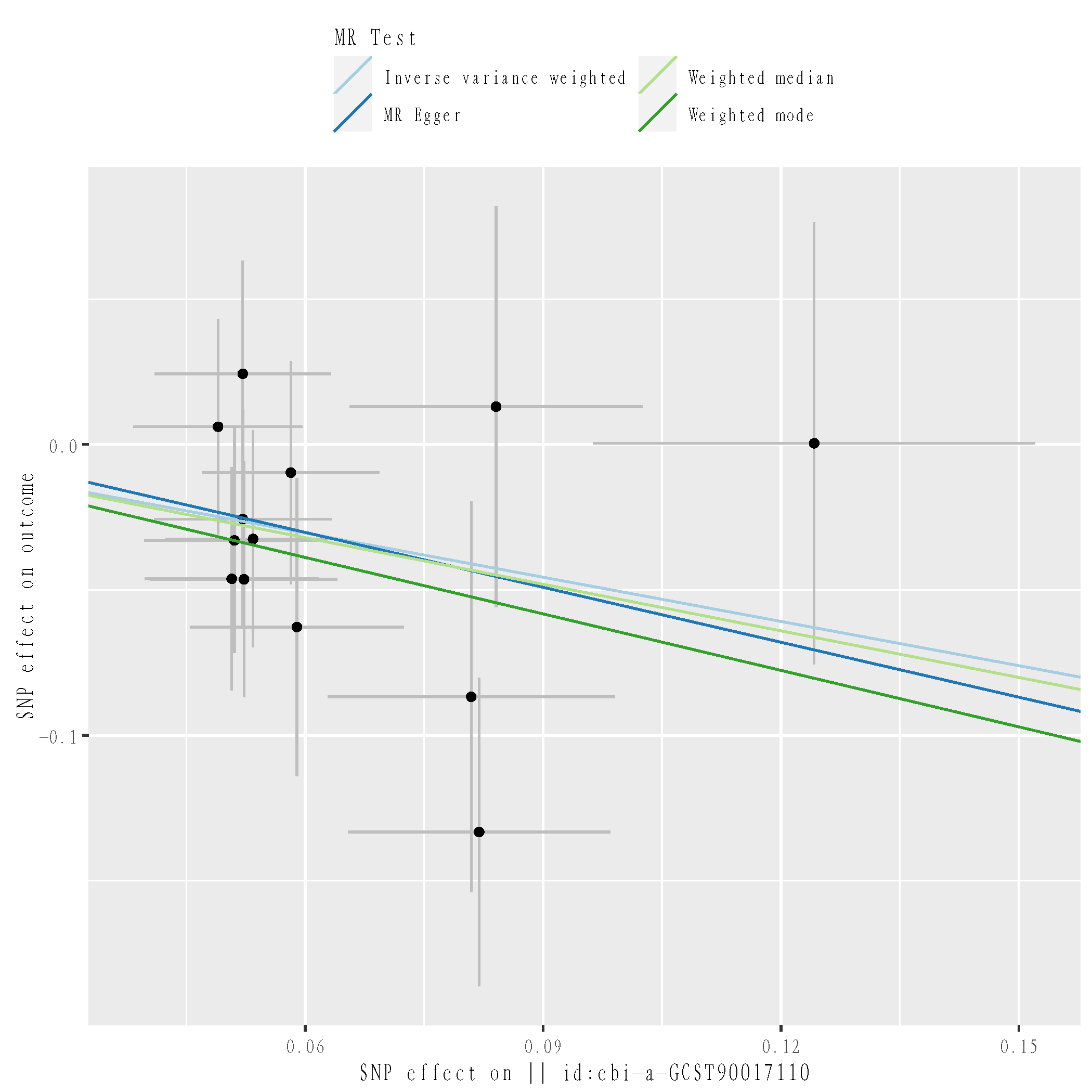


2. The scatter plots of the 4 MR approaches for association between class Gammaproteobacteria and MG.


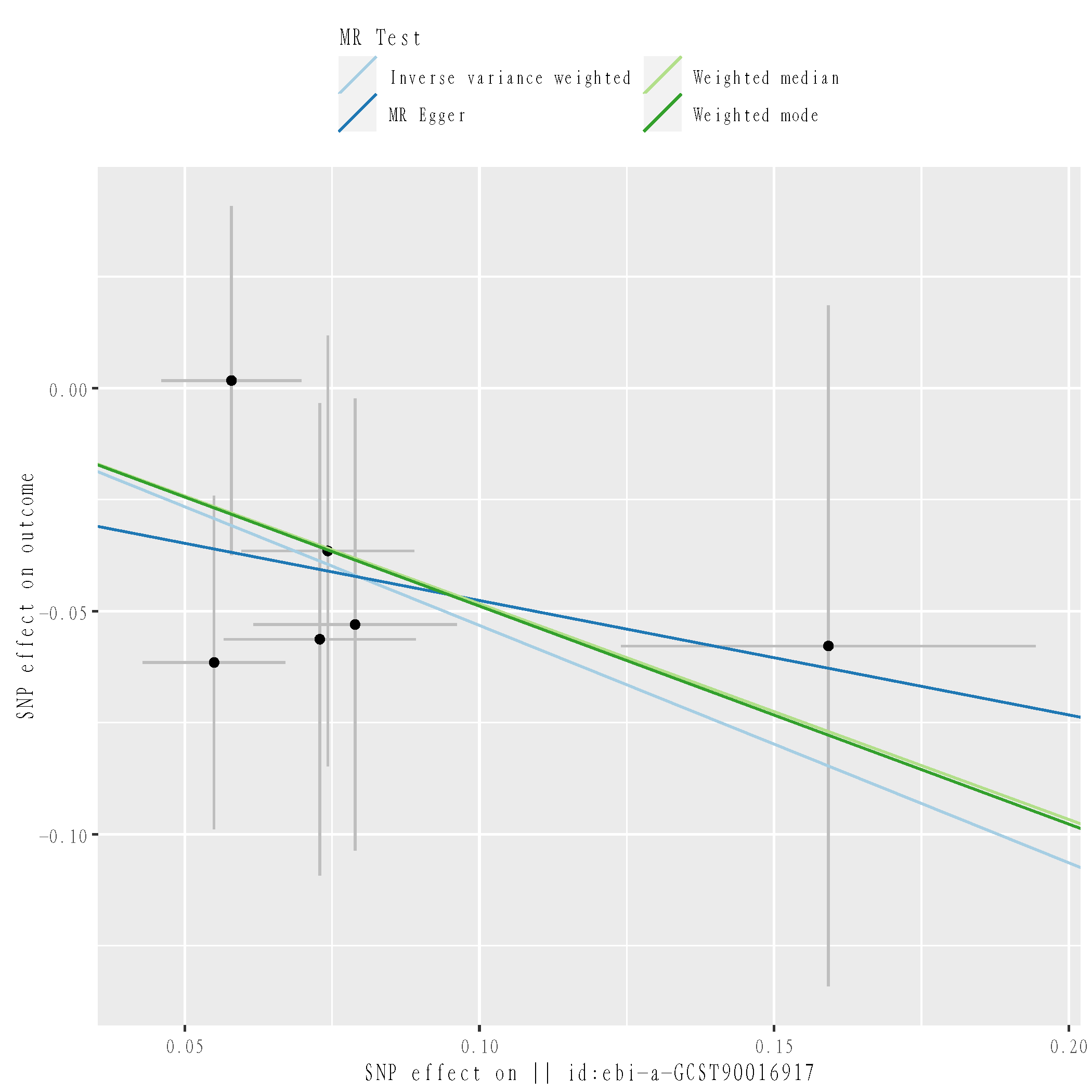


3. The scatter plots of the 4 MR approaches for association between order Mollicutes RF9 and MG.


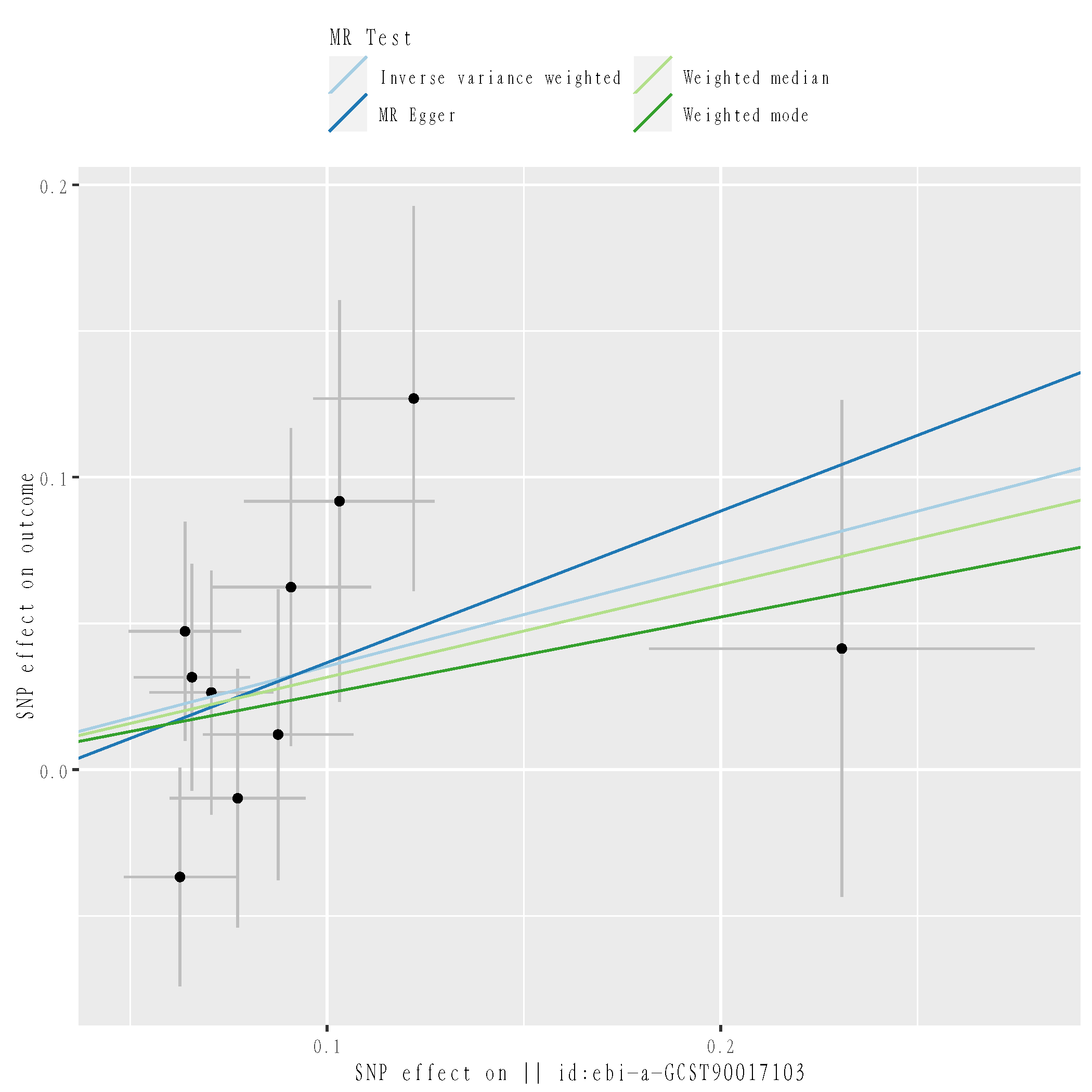


4. The scatter plots of the 4 MR approaches for association between family *Defluviitaleaceae* and MG.


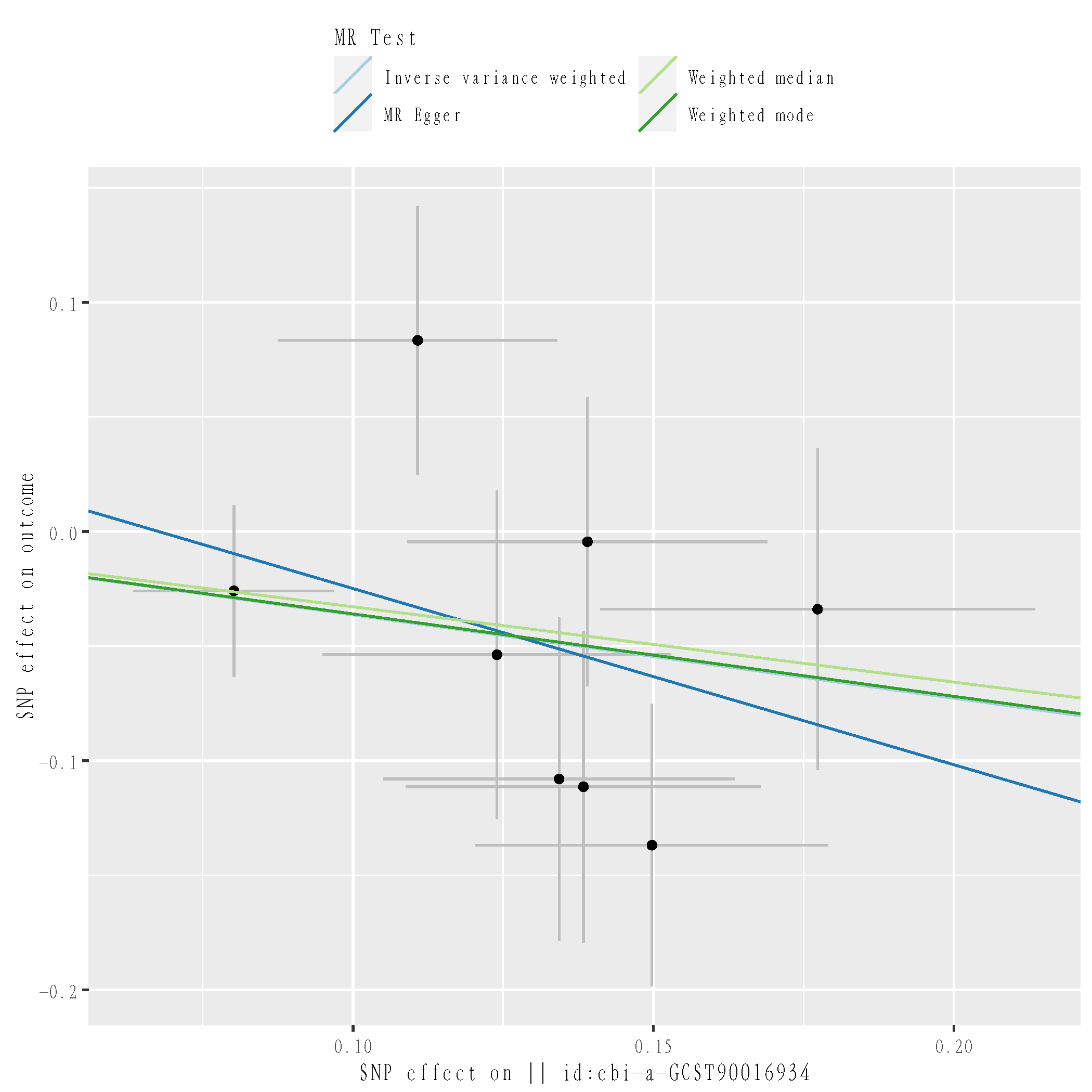


5. The scatter plots of the 4 MR approaches for association between family *Family XIII* and MG.


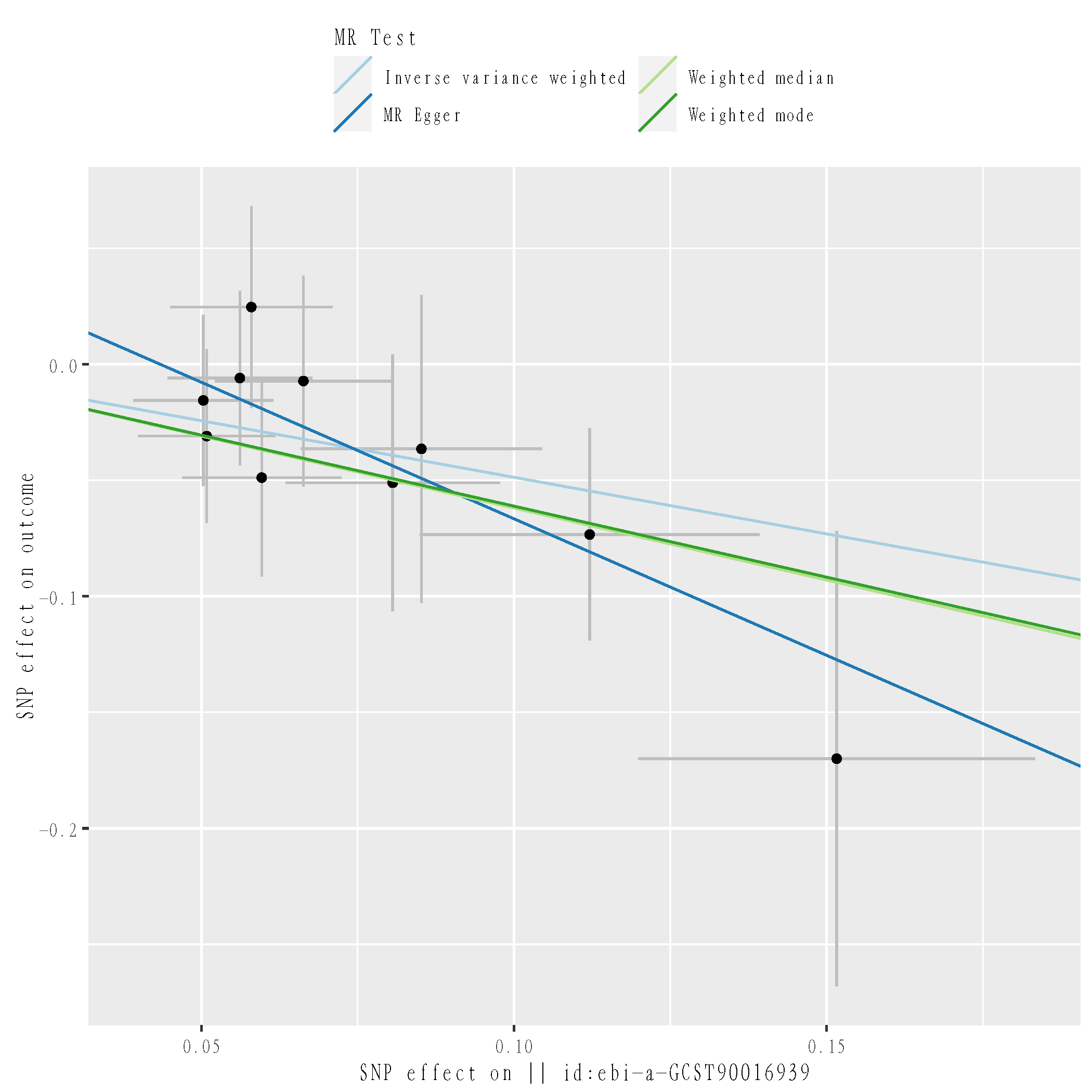


6. The scatter plots of the 4 MR approaches for association between family *Peptococcaceae* and MG.


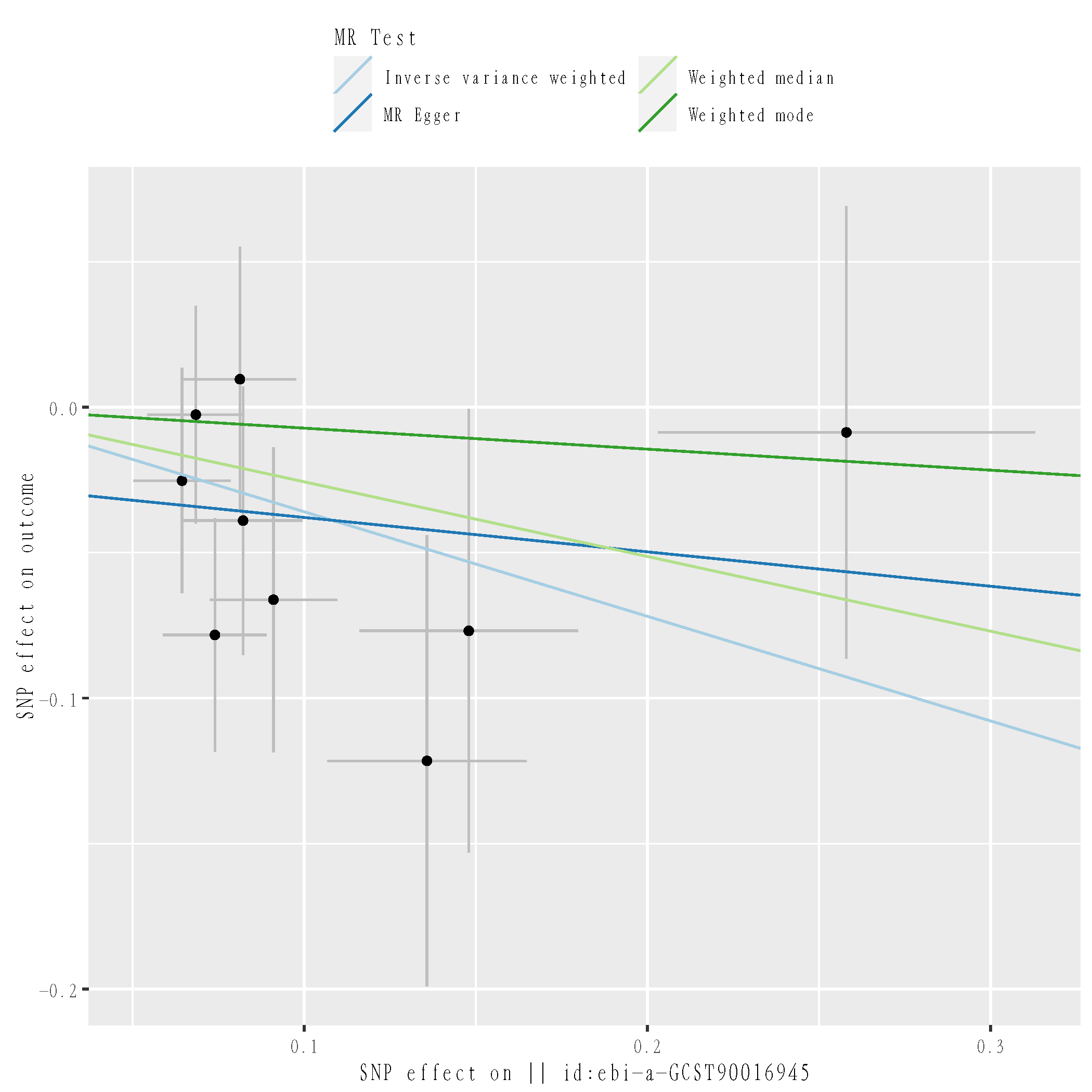


7. The scatter plots of the 4 MR approaches for association between genus *Faecalibacterium* and MG.


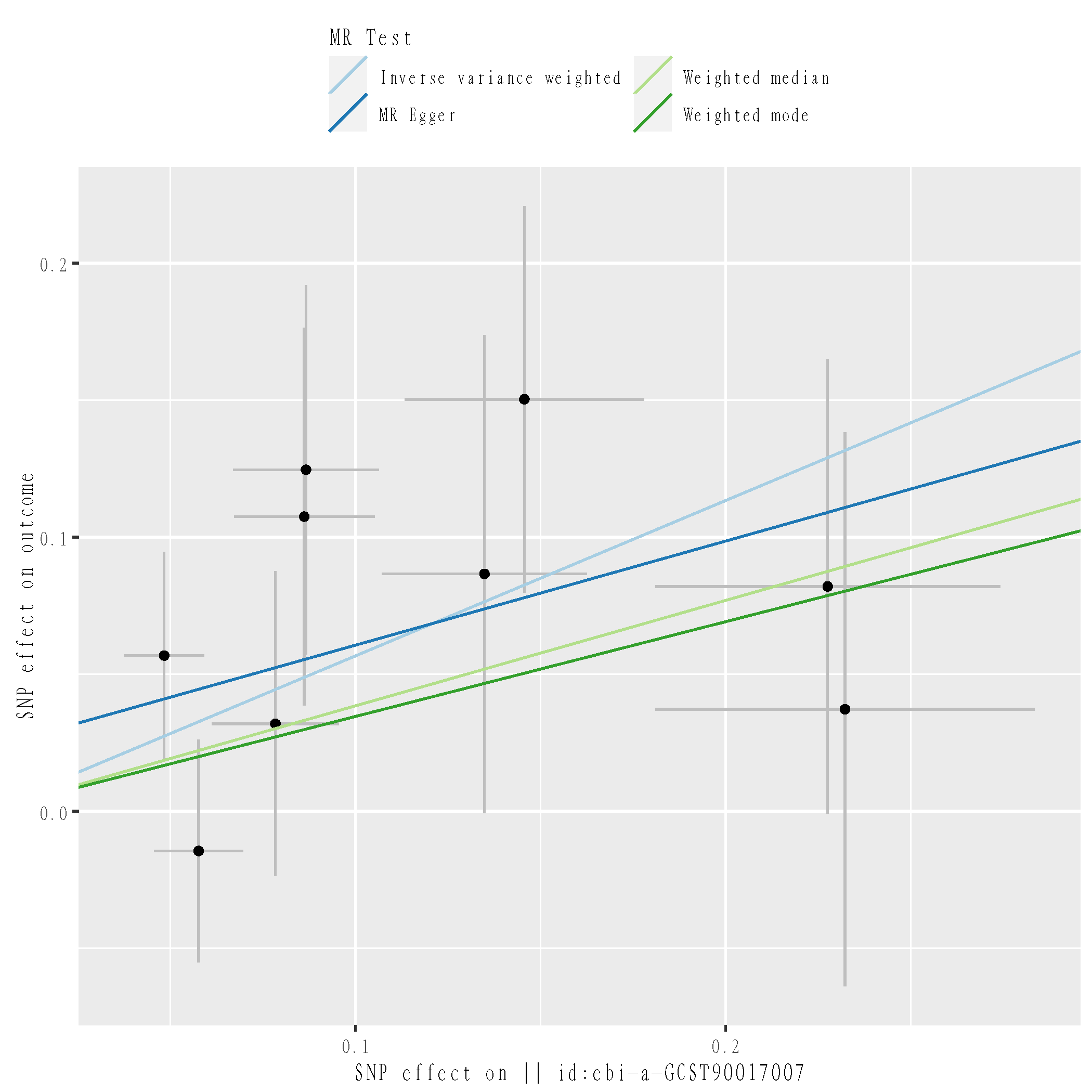


SNP effects were plotted into lines for the inverse-variance weighted test (light blue line), MR-Egger (blue line), weighted median (light green line) and Weighted mode (green line). The slope of the line corresponded to the causal estimation.

**Supplemental Figure S3.**  Forest plots for causal effects of gut microbiota on MG risk with individual SNPs.

1. Forest plots for causal effects of phylum Actinobacteria on MG risk with individual SNPs.


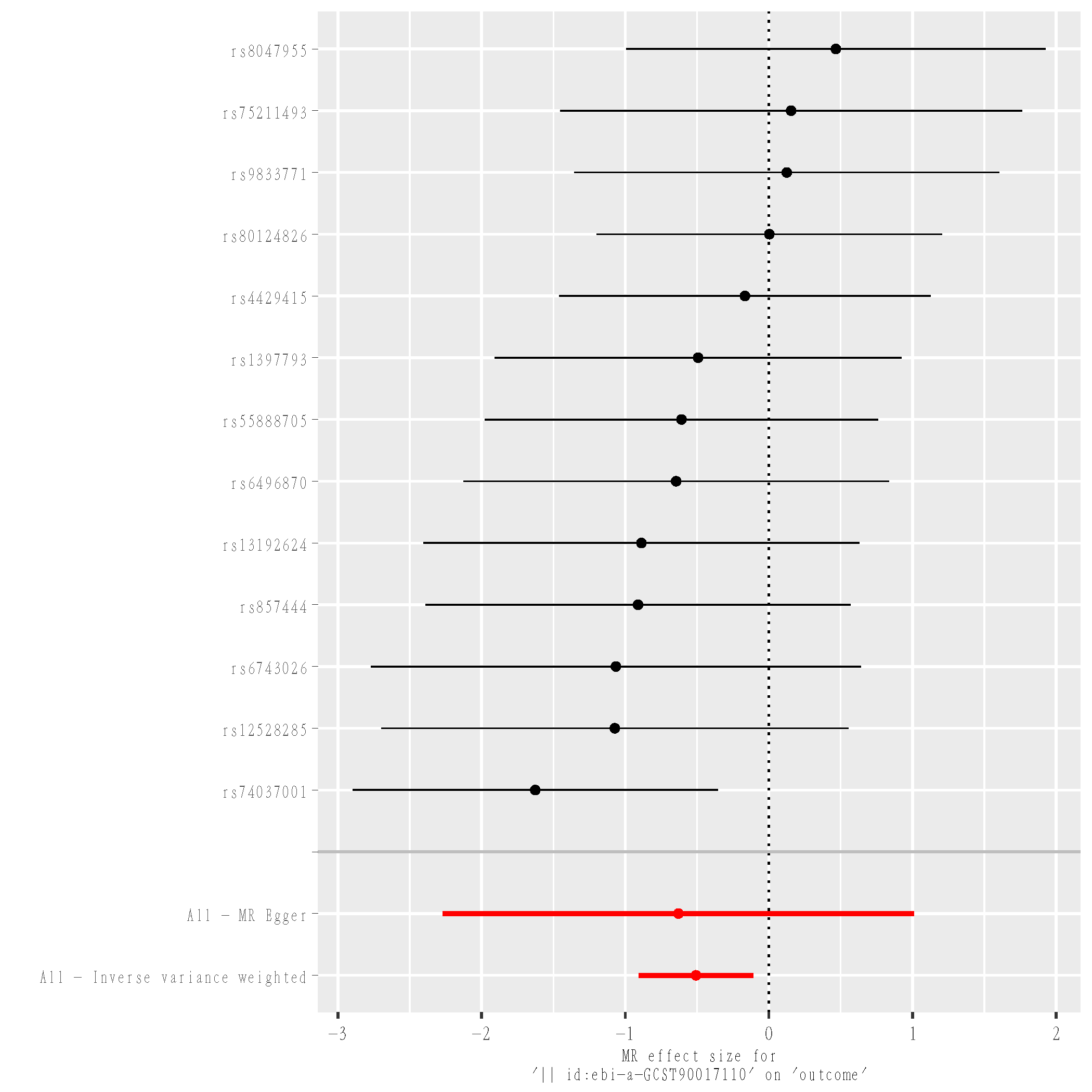


2. Forest plots for causal effects of class Gammaproteobacteria on MG risk with individual SNPs.


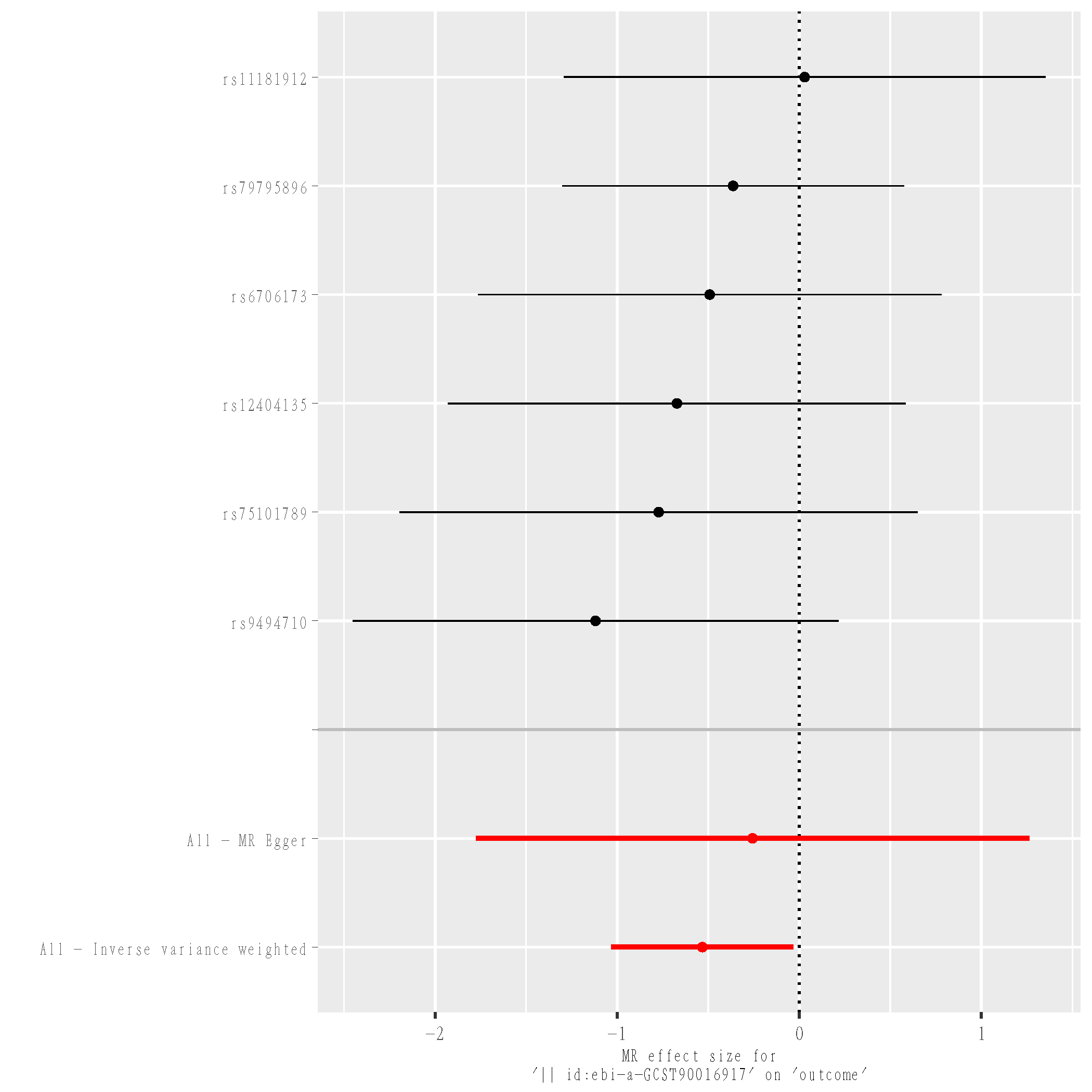


3. Forest plots for causal effects of order Mollicutes RF9 on MG risk with individual SNPs.


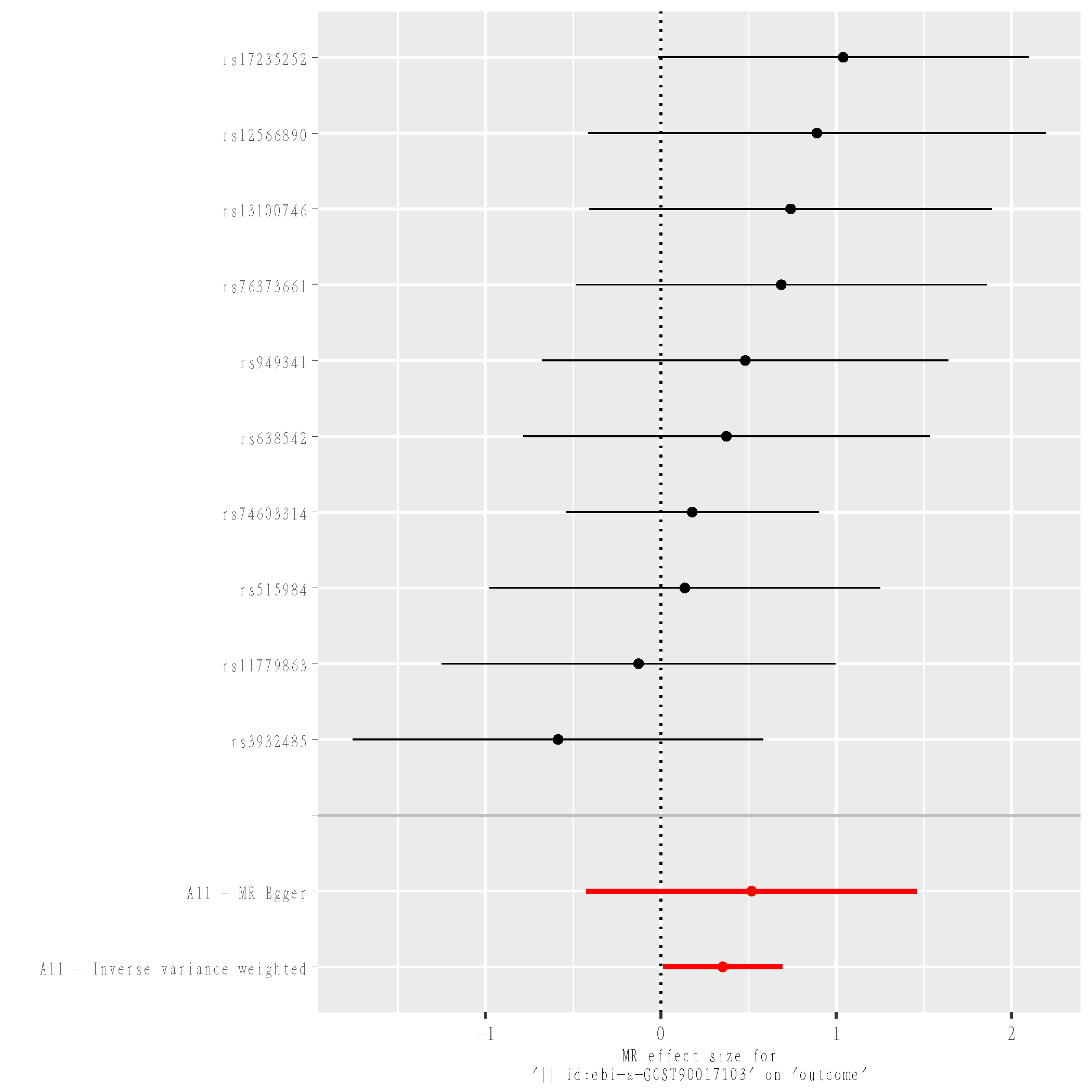


4. Forest plots for causal effects of family *Defluviitaleaceae* on MG risk with individual SNPs.


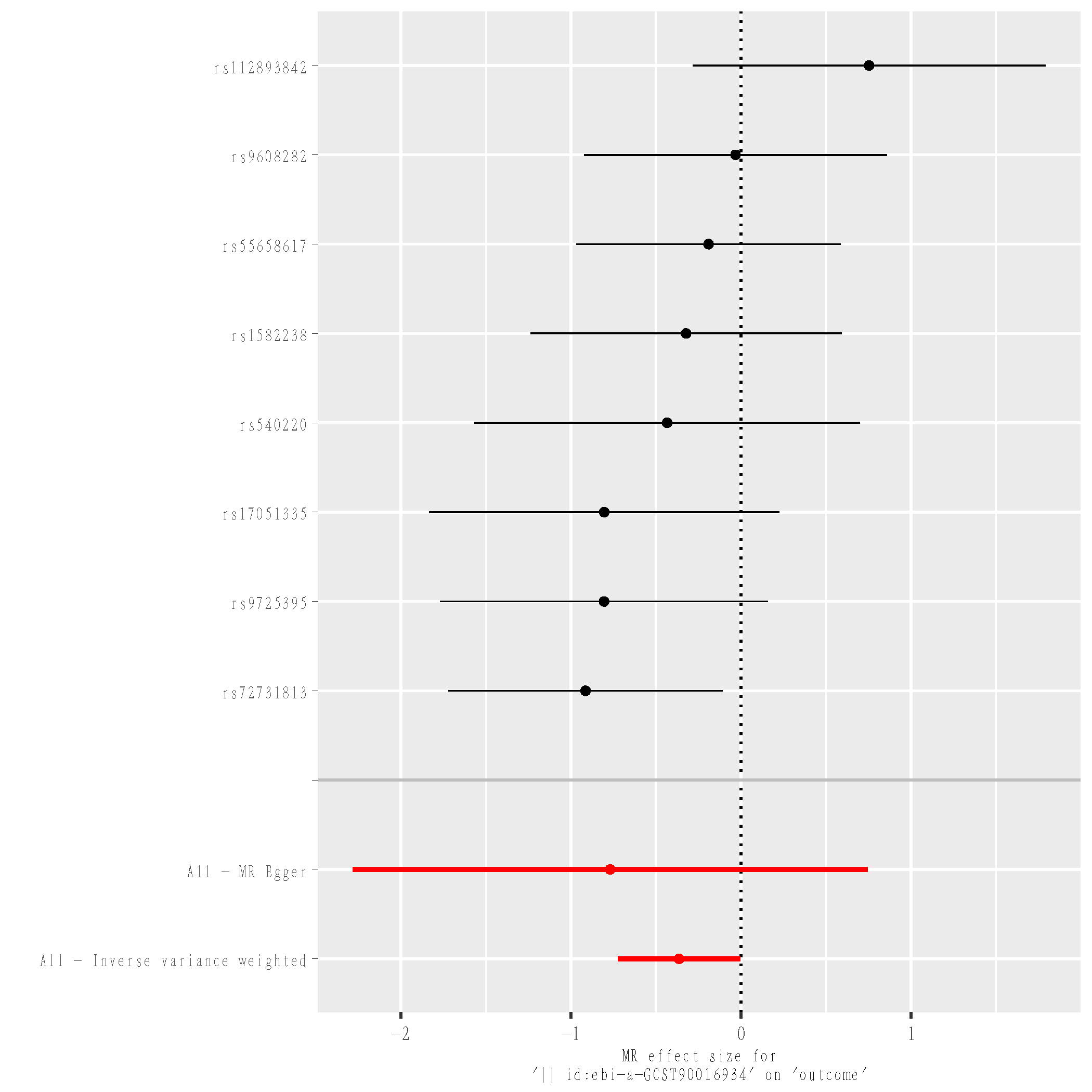


5. Forest plots for causal effects of family *Family XIII* on MG risk with individual SNPs.


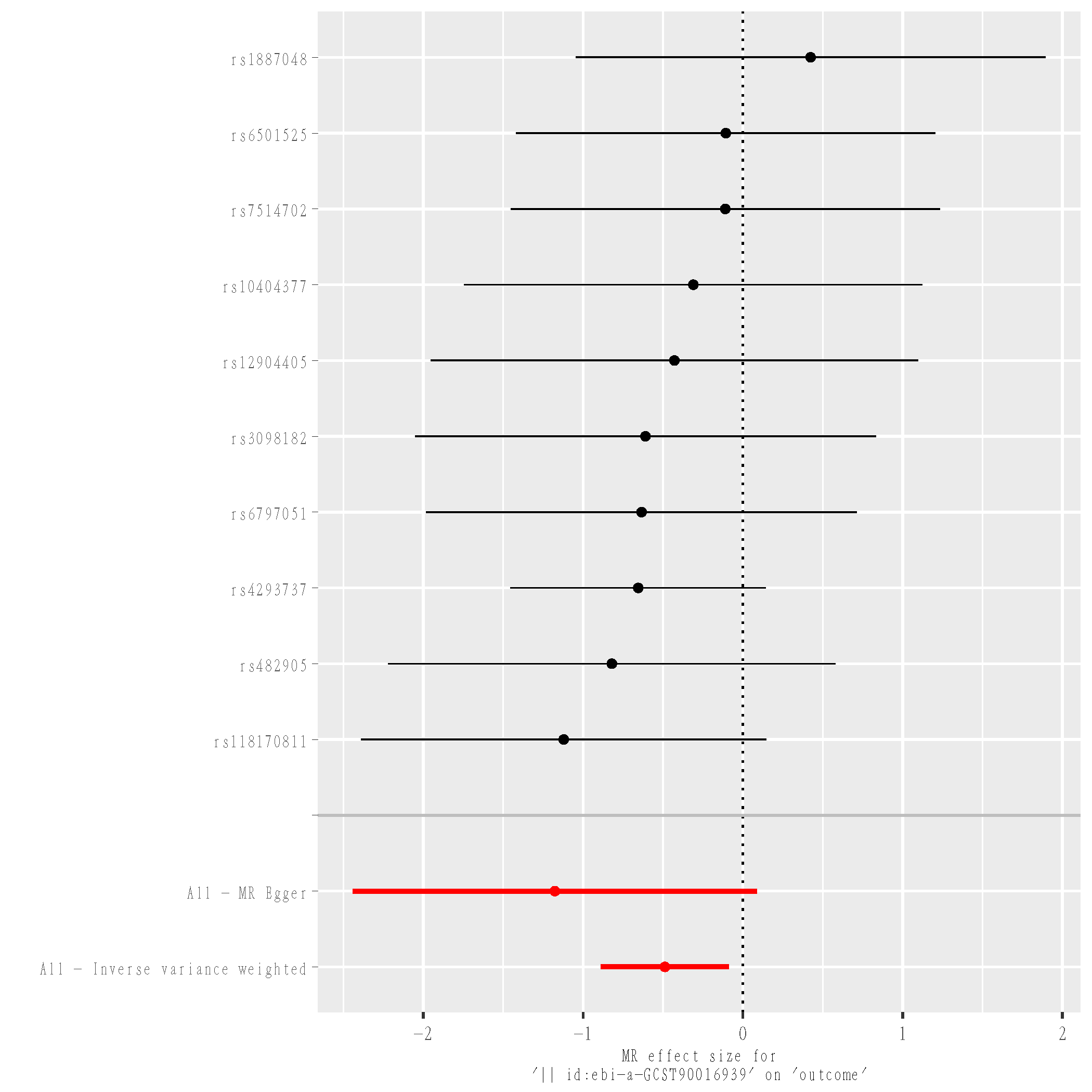


6. Forest plots for causal effects of family *Peptococcaceae* on MG risk with individual SNPs.


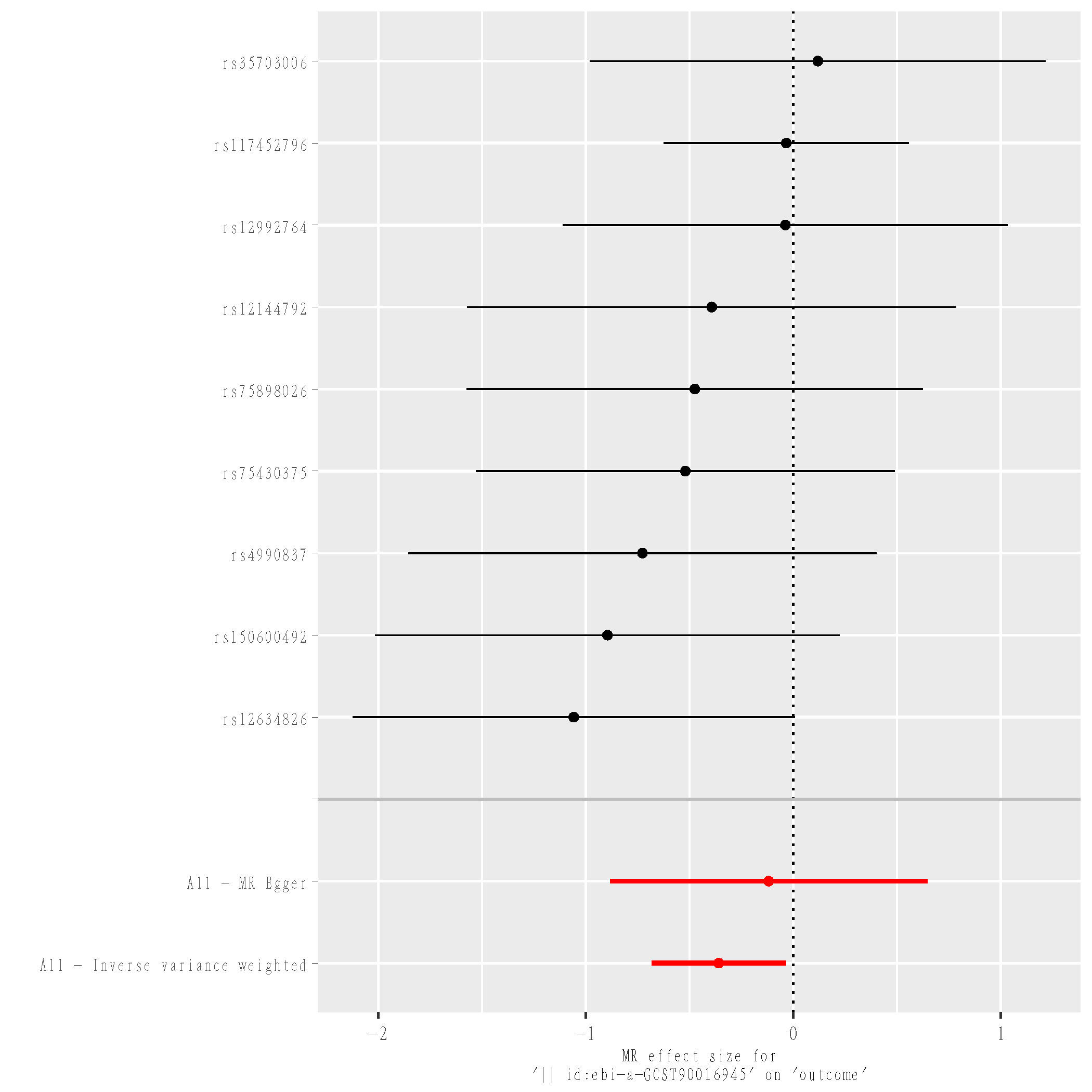


7. Forest plots for causal effects of genus *Faecalibacterium* on MG risk with individual SNPs.


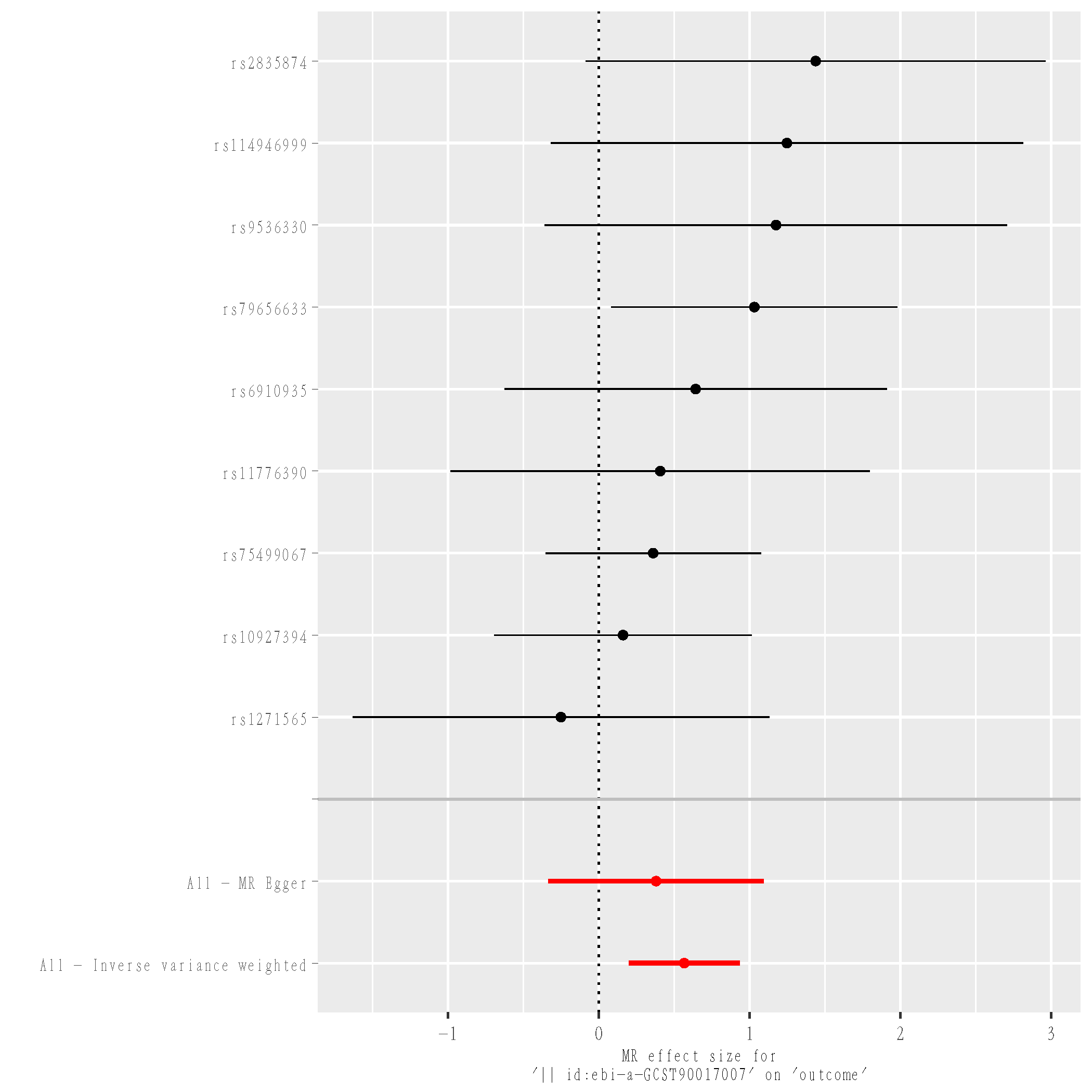


Black points denote the effect estimates of the phylum Actinobacteria, class Gammaproteobacteria, order Mollicutes RF9, family *Defluviitaleaceae*, family *Family XIII*, family *Peptococcaceae* and genus *Faecalibacterium* on the risk of MG using single SNPs, and the black lines signify the 95% CIs of the estimates. The red points symbolize overall effect estimates of the gut microbiota using the Egger and IVW method, and the red lines indicate their 95% CIs**.**
